# Spatiotemporal cytoskeleton organizations determine morphogenesis of multicellular trichomes in tomato

**DOI:** 10.1371/journal.pgen.1008438

**Published:** 2019-10-04

**Authors:** Jiang Chang, Zhijing Xu, Meng Li, Meina Yang, Haiyang Qin, Jie Yang, Shuang Wu

**Affiliations:** College of Horticulture, FAFU-UCR Joint Center and Fujian Provincial Key Laboratory of Haixia Applied Plant Systems Biology, Fujian Agriculture and Forestry University, Fuzhou, China; Peking University, CHINA

## Abstract

Plant trichomes originate from epidermal cell, forming protective structure from abiotic and biotic stresses. Different from the unicellular trichome in Arabidopsis, tomato trichomes are multicellular structure and can be classified into seven different types based on cell number, shape and the presence of glandular cells. Despite the importance of tomato trichomes in insect resistance, our understanding of the tomato trichome morphogenesis remains elusive. In this study, we quantitatively analyzed morphological traits of trichomes in tomato and further performed live imaging of cytoskeletons in stably transformed lines with actin and microtubule markers. At different developmental stages, two types of cytoskeletons exhibited distinct patterns in different trichome cells, ranging from transverse, spiral to longitudinal. This gradual transition of actin filament angle from basal to top cells could correlate with the spatial expansion mode in different cells. Further genetic screen for aberrant trichome morphology led to the discovery of a number of independent mutations in SCAR/WAVE and ARP2/3 complex, which resulted in actin bundling and distorted trichomes. Disruption of microtubules caused isotropic expansion while abolished actin filaments entirely inhibited axial extension of trichomes, indicating that microtubules and actin filaments may control distinct aspects of trichome cell expansion. Our results shed light on the roles of cytoskeletons in the formation of multicellular structure of tomato trichomes.

## Author summary

Forming trichomes is a strategy plants have developed to defend against insects and other stresses. The function of trichomes heavily relies on their morphology, which exhibits high diversity in nature. Unicellular trichomes in Arabidopsis have been used as the model for cell morphological study. But it remains elusive how trichome shapes are established in multicullar trichomes in most plants. Using tomato, we examined multicellular trichome morphology by combining cellular biology approaches and genetic mutants. We found critical roles for of the organization of actin filaments and microtubules in forming multicellular structure of tomato trichomes.

## Introduction

Plant morphogenesis relies on tightly controlled cell division and cell expansion [1, 2]. A number of multi-cellular organs including Arabidopsis roots and pavement cells had been used as the model system to study coordinated morphogenesis [3, 4]. However, the embedment of cells in the tissue makes the cell expansion a converged action from surrounding cells [5]. Trichomes, originating from epidermis protrude from the epidermal surface, form an appending structure which is an ideally simple system for studying the morphogenesis regulation. In Arabidopsis, the trichome is a unicellular structure with three or four branches. Actin filaments control the shape and microtubules are responsible for branching in Arabidopsis [6]. Differently, tomato produces seven morphologically and functionally distinct types of trichomes. Tomato trichomes are all multicellular and generally composed of basal, stalk, and glandular head cells (only in some types) [7]. Thus Tomato trichomes provide an optimal simple model system for studying cell division and cell expansion within a multi-cellular structure.

The cytoskeleton plays an important role in cell expansion and the mechanism has been heavily examined in a wide range of cell types including root and hypocotyl cells, pavement cells, root hairs, pollen tubes, stomata cells and petal cells [3, 8–12]. Transverse alignment of cortical microtubules controls the anisotropic cell expansion in the root and hypocotyl, by directing the deposition pattern of cellulose microfibrils [13]. The jigsaw puzzle-shape of pavement cells is coordinated by localized outgrowth and growth inhibition to form interdigitating lobes and necks (Fu et al., 2002). Transversely arranged cortical microtubules are localized in the neck region have been proposed to restrict cell expansion [3]. In addition to the diffuse expansion mode, some specialized plant cells including root hairs and pollen tubes undergo rapid unidirectional tip growth, which requires the creation of highly specialized cortical fine actin microfilaments configurations [14–16]. A larger number of markers have been used for imaging actin filaments in plants [17–19]. The actin organization revealed by Lifeact marker was shown to faithfully resemble the actin filaments decorated by FIM5 [20, 21]. In polarized cell growth, actin forms dynamic and yet steady fringe structures in the tip of growing tubes, which function as endomembrane guidance, structural support and driving force [20, 22]. Most actin filaments originated from the apical membrane of protruding domain of pollen tubes, arranging into distinct three-dimensional structures to direct vesicle trafficking and accumulation [20, 22–25].

Both cytoskeletons are also required for the hair-like structure of Arabidopsis trichomes. The actin filaments aligned along with the long axis of Arabidopsis trichomes while microtubules were found to align transversely [26]. Disrupt the subunits of ARP2/3 and SCAR/WAVE complexes led to declined fine actin filaments and enhanced actin bundles, which resulted in distorted trichome phenotype [27]. A recent report proposed a model in which ARP2/3 complex directs the actin meshwork to modulate local cell wall anisotropy and maintain tip-biased diffuse growth in Arabidopsis trichomes [28]. Microtubules control trichomes branching by forming microtubule rings in the apical part of the elongating trichome stalk [29]. The *zwicel* (*zwi*) mutant and *fass* (*fs*) mutant display a reduced branching phenotype which is correlated with a defective microtubule organization [30–31]. In cotton fibers, another unicellular trichomes, cytoskeletons were found to similar organization, with actin filaments extending along the long axis while microtubules transversely encircling the fiber shank [32].

However, trichomes in tomato and most other species exhibit different shapes and organizations than Arabidopsis. Tomato trichomes form a protruding cell file that is composed of as many as over ten cells. Based on cell number, morphology and cell identity, tomato trichomes can be classified into functionally diverse types [7]. A previous mutant of SlSAR1 was shown to have defective trichomes, indicating the involvement of microfilaments in morphology of tomato trichomes [33]. However our understanding of genetic and molecular mechanisms behind multicellular trichome formation in tomato remains fragmental.

In this study, we combined genetic screen with live cell imaging approaches to quantitatively study tomato trichome morphogenesis. We found both actin filaments and microtubules exhibited dramatically different patterns in different trichome cells in tomato. Such patterns also changed over developmental stages which could correlate with the distinct cell expansion. Genetic screen for aberrant trichome shapes confirmed the essential role of SCAR/WAVE and ARP2/3 complex in actin organization and trichome morphology. Microtubules appeared to define the anisotropy of the cell expansion while actin filaments seemed to play a major role in the extension of trichome cells. Taken together, our findings provide insights into the role of the cytoskeletons in multicellular trichome morphogenesis.

## Results

### The morphology and organization of tomato trichomes

**Fig 1:**
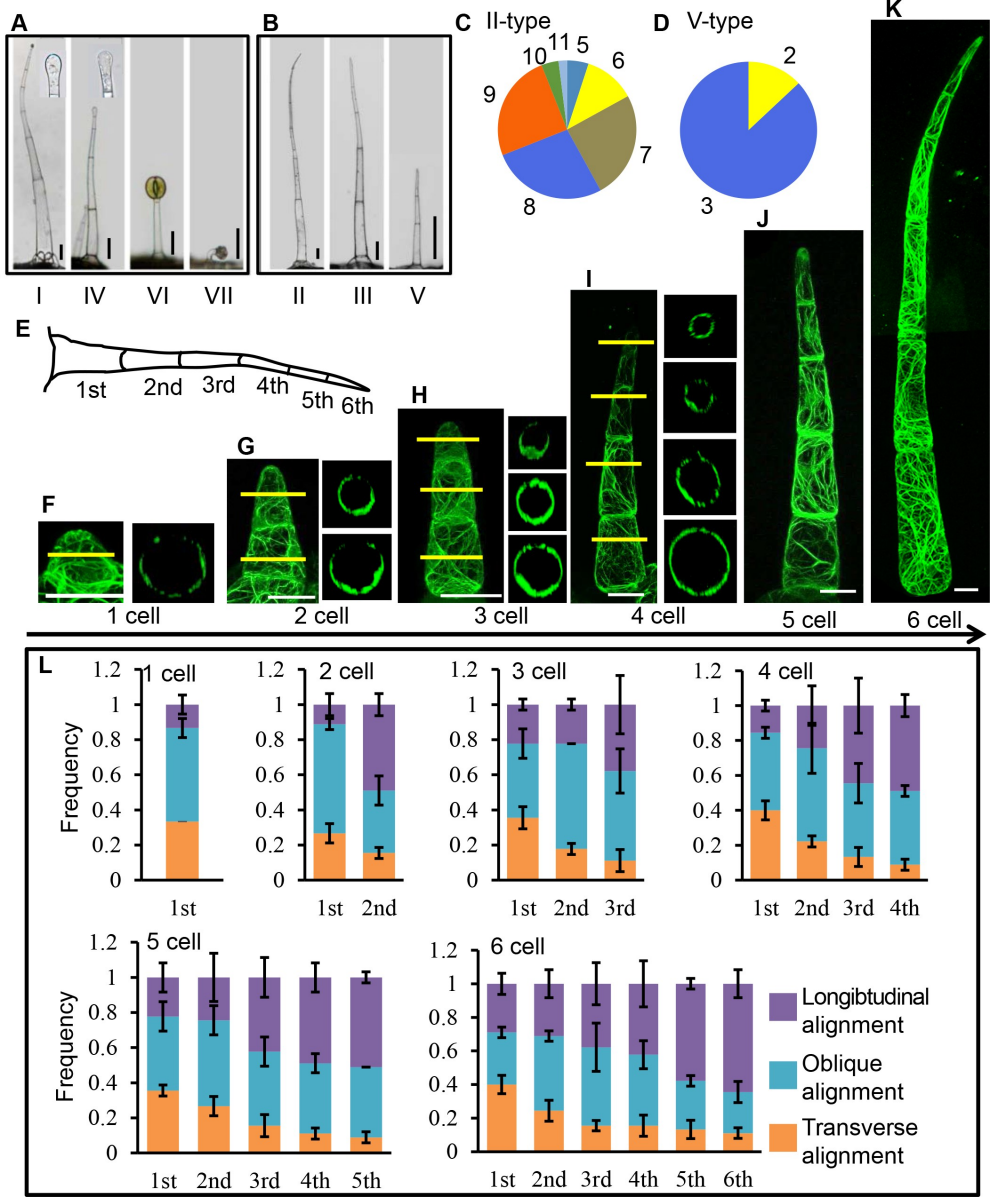
F-actin organization in type II trichomes at different developmental stages. (A) The morphology of four types of glandular trichomes (GT). Bar: 50 μm. (B) The morphology of three types of non-glandular trichomes (NGT). Bar: 50 μm. (C, D) Quantification of cell number in type II and type V non-glandular trichomes. The quantification is based on over 200 trichomes for each type. (E) Schematic diagram of the type II trichome with 6 cells. (F-K) F-actin architecture in type II trichomes at different developmental stages. The right image of each trichome shows the cross section of the lined position in the left image. Bar: 20 μm. (L) The frequency of cortical actin filaments with different orientations. The longitudinal, transverse and oblique alignment represent 0–15°, 75–90°and 15–75°related to the axis of trichome growth respectively.

Unlike unicellular trichome in Arabidopsis, tomato trichomes include a total of seven types of morphologically and functionally distinct trichomes [7]. Among them, there are four types of glandular trichome (GT) and three types of non-glandular trichome (NGT) (Fig 1A and 1B). We first analyzed the abundance of each type of trichome in the stem of tomato (Micro-tom). We found that the type II and V represented two predominant types of NGT (S1A and S1B Fig).

The overall morphology of type II and type V NGT was similar but they consisted of different number of cells (Fig 1C and 1D). Majority of type II trichomes included five to eight cells, with eight cells as the highest ratio (27%) (Fig 1C). Type V trichomes displayed less varied cell number with three cells as the most predominant type (87%) (Fig 1D). The length and width of cells within type II trichome file became gradually reduced along the cell file (S1C–S1E Fig). The average length of the largest basal cell was approximately 728μm which was seven times more than the smallest top cell. But the length of each cell within type V trichome file appeared to be similar despite all cells exhibited tapering shape (S1F–S1G Fig). Most examined type II trichomes still seemed to be in expanding as the cell length variation of each cell position within the cell file is high. In the basal cells, the longest cell was 1141μm in length while the shortest one was only 498μm (S1I Fig). Interestingly, the length of top cells shows wider range, with the longest and shortest ones as 298μm and 44μm respectively (S1I Fig). This suggested that the basal cells might be more mature and many top cells could be newly initiated. Based on the morphological survey, we decided to focus most of our analyses on type II trichome as this type with different cell numbers could represent typical expanding trichome stalk cells.

### Visualization of actin organization at different developmental stages of tomato trichomes

Actin-based apparatus is also implicated in the morphogenesis of unicellular trichome in Arabidopsis [34]. To visualize how actin is spatially organized in different cells of tomato trichomes, we stably transformed tomato with the actin-binding marker Lifeact (p35S:Lifeact-eGFP). Among 18 transformed lines, 10 lines showed normal trichome morphology while the rest had certain level of defective trichome shape and abnormal actin bundles (S2 Fig). The ten normal lines were then chosen for further live-cell imaging analyses. To visualize different developmental stages of type II trichomes, we imaged young leaves (14 days after germination) and identified trichomes as early as one-cell and initiating stage (Fig 1F–1K). At one-cell stage, up to 86% actin filament showed transverse (0–15 degree against the horizontal plane) or oblique (15–75 degree) alignment, and the longitudinal actin filaments were only 14% (Fig 1F and 1L). However, the actin alignment in this cell seemed to be spatially heterogeneous, with transverse actin filaments girdled at the conjunction base and longitudinal actin aligned beneath the protruding dome (Fig 1F).

As the cell number increased, type II trichomes exhibited distinct actin patterns in different cells. The basal cell maintained around 80% transverse or oblique actin filaments while these alignments gradually reduced and longitudinal actin filaments progressively increased upward along the trichome (Fig 1F–1L). At 6-cell stage, longitudinal actin filaments became over 60% in distal top cells (Fig 1L). These observations suggested that actin filaments rearranged during the elongation of tomato trichomes. Interestingly, almost all trichome cells including the initiating one-cell stage one appeared to be highly vacuolized during all developmental stages as most actin tended to assemble into cortical network (Fig 1F–1I).

**Fig 2:**
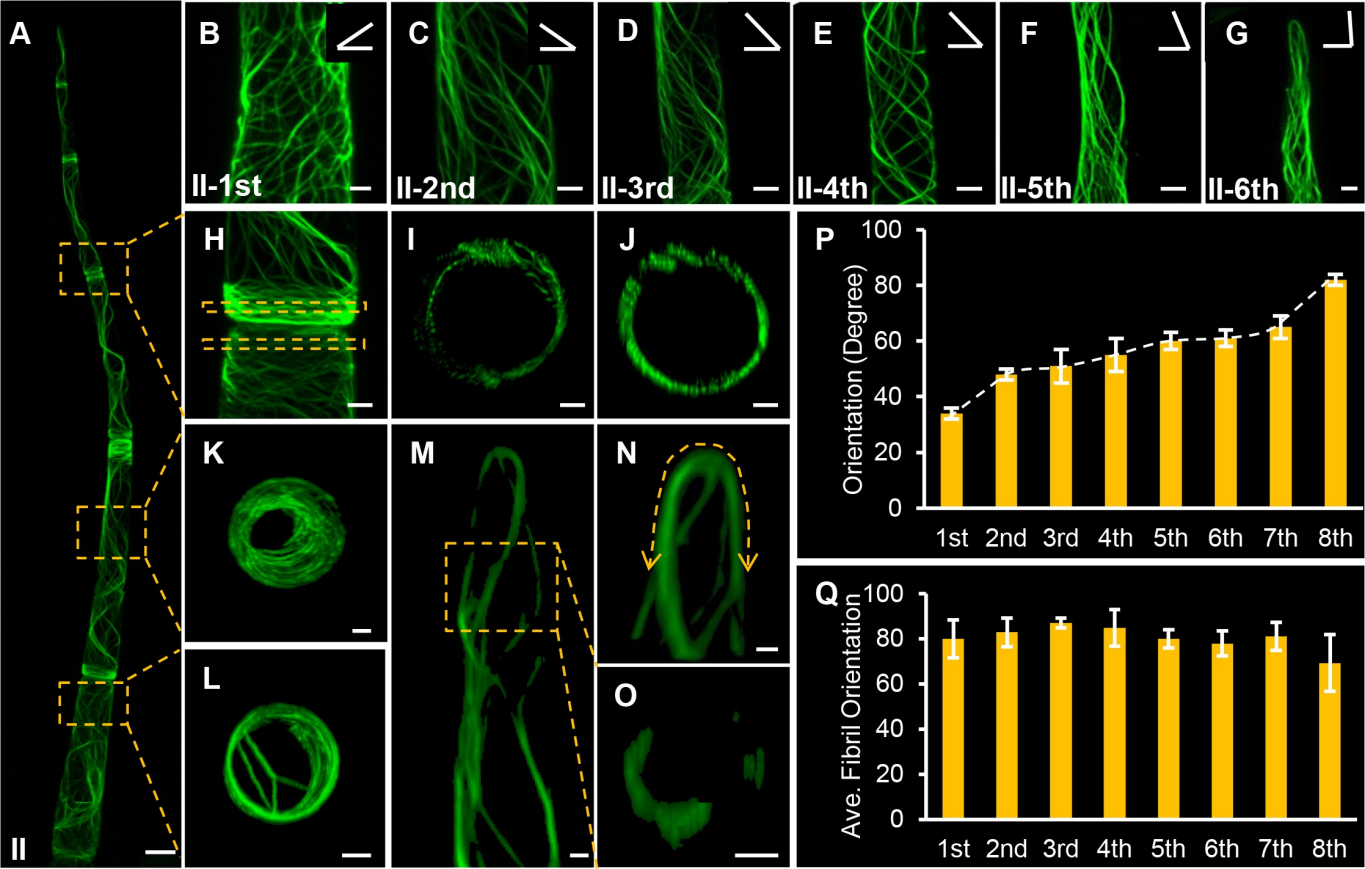
Visualization of actin organization in type II trichomes using Lifeact-eGFP fusion protein. (A) A panoramic micrograph of actin organization in the type II trichome cell file. The boxed areas are zoomed in H-L. Bar: 50 μm. (B-G) Zoomed view of actin arrangement in each cell of the type II trichome. The cell order is marked in the individual micrograph. The angle diagram showed in the up right corner of each image represents the average angle of actin filament in each cell. Bar: 10 μm. (H) Zoomed view of the position in the corresponding box in (A). There are transverse actin filament meshes at the end of cell. Bar: 10 μm. (I, J) The cross section of the positions boxed in (H) respectively. The actin filaments are organized in the cortical region. Bar: 10 μm. (K, L) The cross section of the position in the corresponding box in (A). Bar: 10 μm. (M) Projected confocal micrograph of the actin configuration in the top cell of type II trichome. Bar: 10 μm. (N) Zoomed view of the actin arrangement in the dome region of the type II trichome cell file. A double arrowhead dotted line presents the actin filaments loop across the whole dome cap in the top cell. Bar: 10 μm. (O) The cross section of the position in the corresponding box of (M). Bar: 5 μm. (P) Quantification of cortical actin filament orientation in type II trichome cells. The angles are relative to the direction perpendicular to the axis of the growth. (Q) Average actin filament orientation of type II trichome cells measured by FibrilTool.

When the trichome reached six-cell stage, most cells further elongated and actin filaments exhibited the overall spiral arrangement along the long axis (Fig 2A). We next quantitatively measured the orientation using FibrilTool (Fig 2P). Interestingly, the extent of helix appeared to be progressively reduced from the basal cell to the distal cells at the top. In the first basal cell, actin filaments aligned approximately 34 degree against the direction perpendicular to the axis of the growth, while this angle gradually increased along the cell file and eventually formed the longitudinally actin bundles (Fig 2B–2G and 2Q). Similar to type II trichomes, actin filaments exhibited spiral-to-longitudinal pattern in type V trichomes (S3 Fig).

The longitudinal actin arrangement in the distal top cells physically resembled the actin bundles in the shank of tip growth cells such as pollen tubes and root hairs. However, actin filaments often formed a doom-shaped actin cap at the protruding head of the top cells, suggesting it might be a different mechanism from the typical tip-growth (Fig 2M and 2N). Within most cells, the actin arranged into transversely aligned mesh on basal and apical sides, while actin in the middle part of the cell showed more helical alignment (Fig 2H). Similar to the younger stages, most six-stage trichome cells were highly vacuolized as evidenced by the cortical actin organization (Fig 2I–2L and 2O). It is possible that this non-uniform organization of actin within a cell reflect the putative distinct expansion rate of the cell surface.

### The dynamics of actin filament in different trichome cells

**Fig 3:**
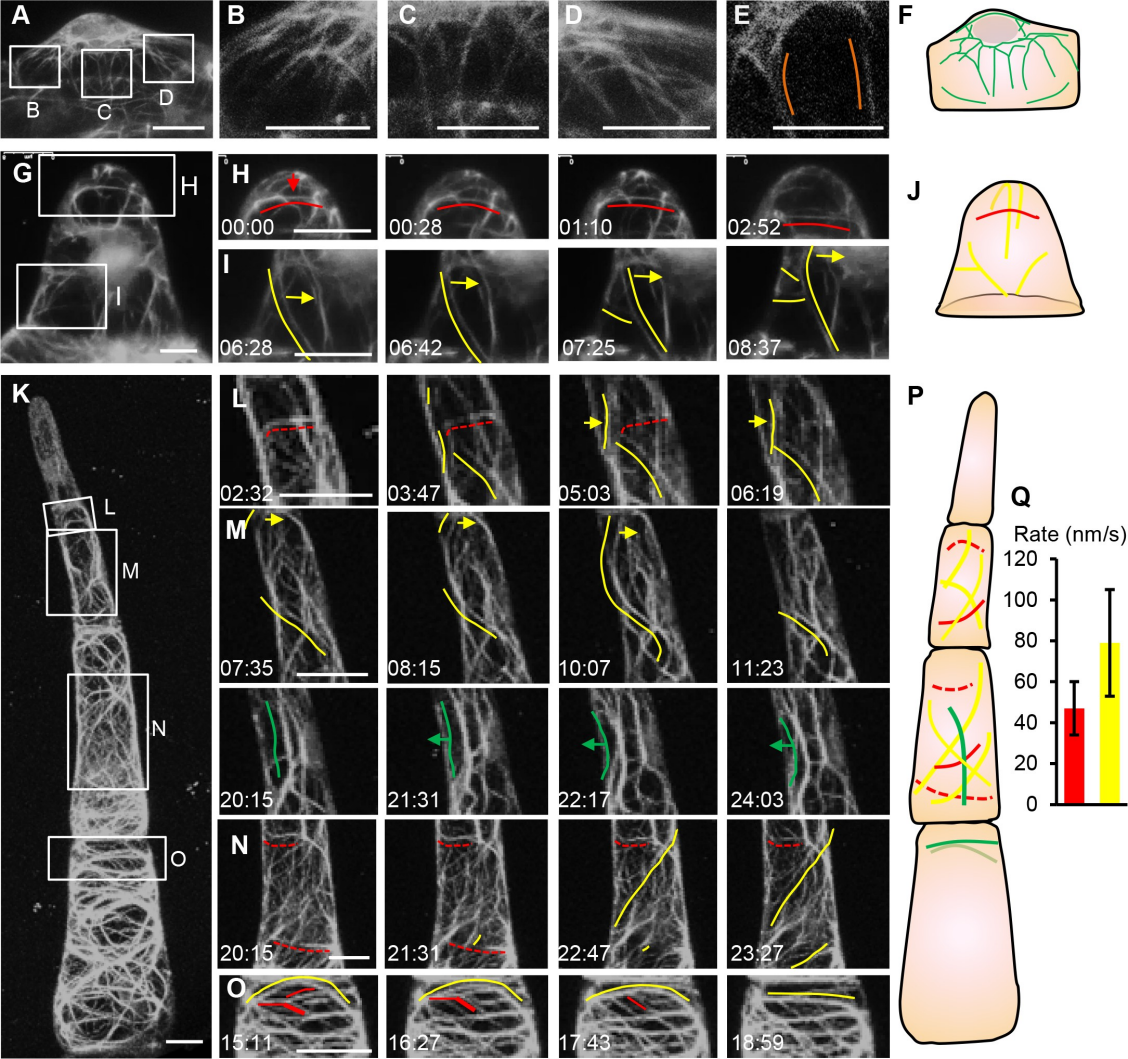
Dynamics of actin filament at different developmental stages. (A-F) The organization of actin filaments during trichome initiation. Bar: 15μm. (B-D) are zoomed view of boxes in (A). Bar: 10μm. (E) The longitudinal section of A. The orange lines show the actin filaments in the cytoplasm. Bar: 10μm. (F) The schematic diagram shows the trichome initiation process. The green lines show the actin filaments; the gray oval shows the nucleus. (G-J) The actin dynamics at the one-cell stage. The red lines show the transverse actin filaments shifting down. The yellow lines show the oblique actin filaments in moving. The arrow indicated the movement direction of the actin filaments. (H and I) are zoomed view of boxed regions in the G. (J) shows the schematic diagram of the one-cell stage trichome and the dynamics of the actin filament. Bar: 5μm. (K-Q) The actin filaments at the four-cell stage. The red dotted lines show the depolymerization of transverse actin filament. The yellow lines show the oblique or helix actin filaments. The green line indicated the actin bundles. (L-P) were zoomed view of boxed region in K. (P) is the schematic diagram of four-cell stage trichome. Bar: 10μm. (Q) The depolymerization rate of the transverse actin alignments (red bar) and the polymerization rate of the longitudinal and oblique actin filaments (yellow bar) in the second and third cell of the trichomes at the four-cell stage.

To understand how distinct actin patterns form in different trichome cells, we performed time-lapse imaging in trichomes at initiation, one-cell and four-cell stages (Fig 3 and S1–S3 Videos). When a trichome started to initiate from epidermis, the nuclear moved into the bulging site and actin filament formed a cap-like mesh under the protruding doom (Fig 3A–3F and S1 Video). In the upper part of the cell, almost all actin filaments in the cytoplasm appeared to be longitudinal (Fig 3E and S1 Video). At the one-cell stage, we found that the actin filaments were rarely depolymerized and often moved up and down or left and right (Fig 3G–3J). Meanwhile, longitudinal actin filaments sometimes formed transverse or oblique branches (Fig 3I). As the four-cell type II trichomes represented the intermediate stage of the trichome development (Fig 3K–3Q), we thus chose this stage for further dynamics analyses. In top cells, we observed high frequent depolymerization of transverse actin filament (Fig 3L and 3N and S3 Video). In contrast, longitudinal or oblique actin filaments tend to polymerize or only shifted left and right (Fig 3M, 3N and 3P). In the basal cell, transverse actin filaments were dominant and most transversely aligned actin appeared to be less dynamic (Fig 3O). In this cell, oblique actin filaments tended to have higher frequency of polymerization to form actin cable (Fig 3O). These observations indicated distinct actin patterns in different trichome cells formed through regulating the dynamics of differentially oriented actin filaments.

### Organization and dynamics of microtubules in tomato trichomes

**Fig 4:**
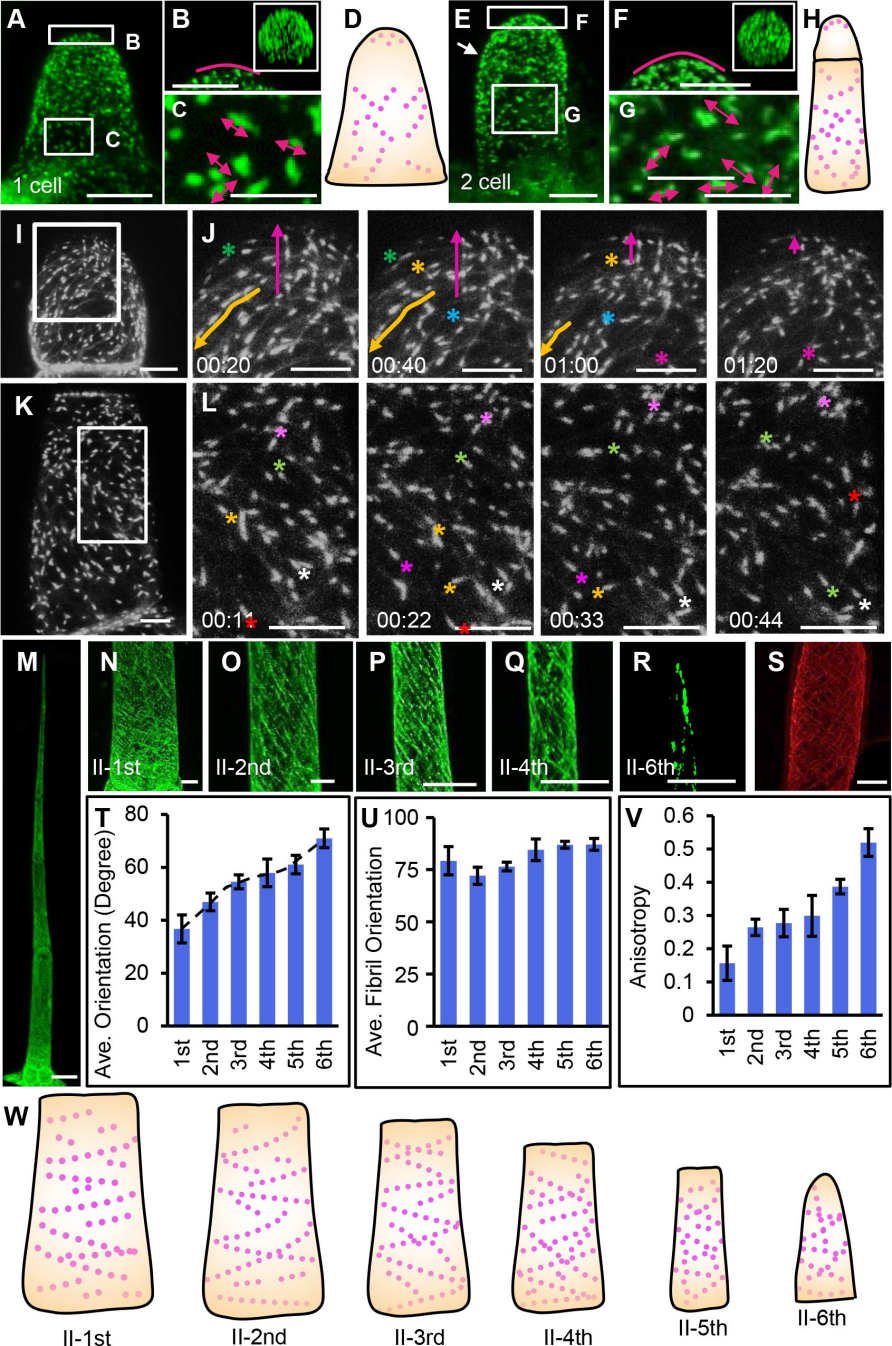
Visualization of EB1a-eGFP fusion protein in type II trichomes. (A)Overall view of the signal of EB1a-eGFP at the one-cell stage. Bar: 5 μm. (B C) Zoomed view of EB1a-eGFP in box regions in A. The zoomed image on the upper right in B shows the corresponding cross-section. The red curved line in B indicated the apical zone of the cell. The red arrows in C show the possible direction of the moving EB1a-GFP. Bar: 2.5 μm. (D) The schematic diagram shows EB1a-eGFP at one-cell stage. The red dots indicate the signal of EB1a-GFP. (E) Overall view of the signal of EB1a-eGFP at the two-cell stage. The white arrow points to the cell division line of the two cells. Bar: 5 μm. (F G) The magnified image of the organization of EB1a-eGFP in the boxed region of E. The zoomed image on the upper right in the (F) shows the corresponding cross-section. The red curved line in the (F) indicated the apical zone of the cell. The red arrows show the possible direction of the moving EB1a-GFP. Bar: 2.5 μm. (H) The schematic diagram shows EB1a-eGFP at two-cell stage. The red dots indicate the signal of EB1a-GFP. (I J) The EB1a-eGFP dynamics of the top cell at the two-cell stage. (J) is zoomed view of boxed region in K. The color asterisks and arrows show the traceable EB1a-eGFP. Among them, the purple arrows show the GFP signal moving to the tip of the cell. Bar:5μm. (K L) The EB1a-eGFP dynamics of the basal cell at the three-cell stage. (L) is zoomed view of boxed region in K. The color asterisks show the traceable EB1a-eGFP. Bar: 5μm. (M) Overall view of the signal of EB1a-eGFP in type II trichomes. Bar: 50 μm. (N-R) zoomed view of the signal of EB1a-eGFP in each cell of type II trichomes. The position of the cell was marked in the bottom left corner of each image. B-G, bar: 10 μm. (S) Immuno-staining image of microtubules in type II trichomes in WT using anti-tubulin. (T) Average direction of EB1a-GFP movement in type II trichomes measured by Image J. The angles are relative to the direction perpendicular to the axis of the growth. (U) Average fibril orientation of EB1a-GFP movement in type II trichomes measured by FibrilTool. (V) Quantitative analyses of the anisotropy of the direction of EB1a-GFP movement by FibrilTool. Anisotropy values range from 0 to 1. 0 indicates pure isotropy, and 1 represents pure anisotropy. (W) The schematic diagram of EB1a-eGFP at the different cell of the type II trichome. the purple dotted line shows the orientation of microtubule.

During the formation of unicellular trichome in Arabidopsis, microtubules are mostly involved in trichome branching [6, 35]. To understand how microtubules regulate trichomes in tomato, we performed live imaging of microtubule organization by visualizing stably transformed tomato expressing end-blocking protein (EB1a) driven by CaMV35s promoter. There were no visible phenotypes in transgenic plants and we could see clear eGFP fluorescence in trichomes of the transgenic plants (S4A–S4D Fig). We first looked the microtubule organization in trichomes at different developmental stages. At one-cell stage, EB1a-eGFP showed as dots spread at the tip of the cell, and the EB1a-GFP seemed to cover the whole dome of trichomes (Fig 4A–4C). In the basal part of the cell, EB1a-eGFP displayed a comet shape, a sign of rapid moving track during the confocal scanning (Fig 4A–4C). By tracking the comet shape, we could infer the direction of microtubule movement. In the basal part of cells, many EB1a-GFP appeared to traffic spirally (Fig 4D). At two-cell stage, there were also two groups of EB1a signal, with less dynamic ones on the membrane of protruding dome and quickly moving ones in the basal cell of the trichome (Fig 4E and 4F). The time-lapse imaging showed that EB1a-GFP frequently moved to the tip of the trichomes (Fig 4I and 4J and S4 Video). In the basal cell, we could easily detect the comet shaped EB1a-eGFP (Fig 4E–4H). Further live time-lapse imaging in the basal cell of the three-cell stage showed that the comet EB1a-GFP moved spirally up or spirally down (Fig 4K and 4L). At the six-cell stage, EB1a in trichome cell files showed an overall helical movement pattern (Fig 4M and 4W). Further immunofluorescence imaging using anti-TUB antibodies in observed cells (many other cells were lost due to the specimen processing) displayed the similar spiral pattern as shown by EB1a-eGFP (Figs 4S and S4G). Interestingly, the movement pattern of EB1a displayed a similar angel transition as actin filaments (Fig 4N–4W).

### *dt* mutants distinctly affect different trichomes cells

**Fig 5:**
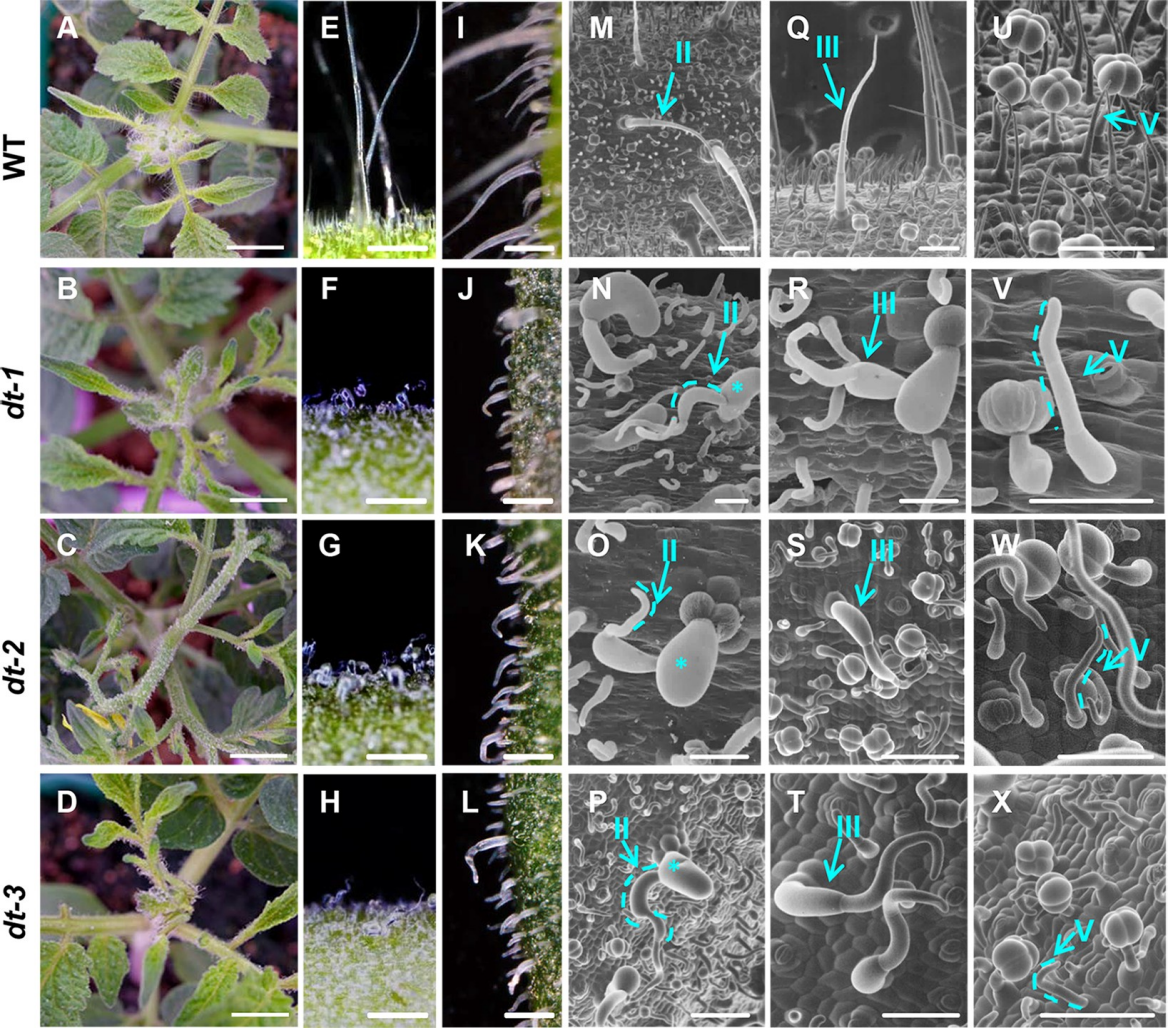
Phenotype of *distorted trichome* (*dt*) mutants. (A, E, I, M, Q, U) WT; (B, F, J, N, R, V) *dt-1* mutant; (C, G, K, O, S, W) *dt-2* mutant; (D, H, L, P, T, X) *dt-3* mutant. Bar: 1mm. (A-D) Phenotypes of WT and *dt* mutants. Bar: 1cm. (E-L) Stereoscopic microscopes of trichomes on the stem (E-H) and the edge of the leaf (I-L). Bar: 1 mm; I-L, Bar: 250 μm. (M-X) Trichome phenotype of WT and *dt* mutants under SEM. Type II, Type III and Type V trichomes are marked. The anisotropic expanding cells are marked by asterisks. The dotted lines outline bending cells. Bar: 100 μm.

**Fig 6:**
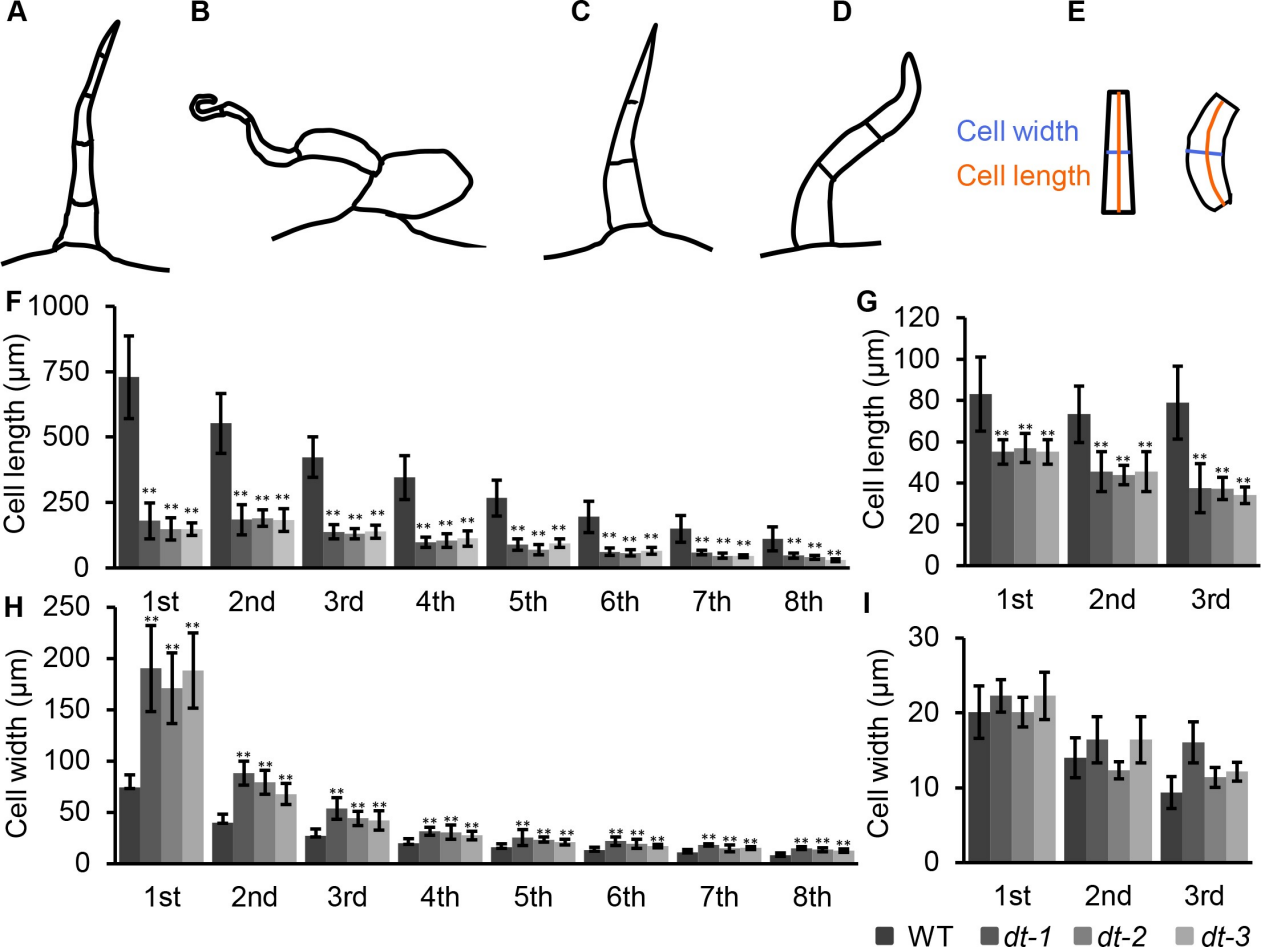
Quantitative comparison of trichome phenotypes between WT and *dt* mutants. (A, B) Schematic illustration of type II in WT (A) and *dt* mutants (B). (C, D) Schematic illustration of type V trichomes (C) in *dt* mutants (D). (E) Schematic illustration of the cell length and width measured in this analysis. (F, H) Cell length and width of type II trichomes of WT and *dt* mutants. (**P<0.01) (G, I) Cell length and width of type V trichomes of WT and *dt* mutants. (**P<0.01).

To uncover the genetic and molecular mechanisms controlling the cell expansion of tomato trichomes, we examined EMS mutagenized lines. We identified six mutants with visibly aberrant trichome morphology and named them as *distorted trichomes-1~6* (*dt-1~6*) (Fig 5A–5D; *dt-4~6* were not displayed). Compared with wild type in which trichomes were straight and perpendicular to the epidermal surface, the trichomes on stems and leaves of all mutants were curly and prostrated under stereomicroscope (Fig 5E–5L). The phenotypes of mutant trichomes were more prominent under scanning electron microscopy (SEM). The trichomes of WT were a group of cells with tapered diameter that were connected end to end. However, the first basal cell of type II trichomes in mutants became dramatically swollen in one direction while the middle cells bent with little swollen, and the top cell curved into a hook shape (Figs 5M–5X and 6A and 6B).

To better understand the morphological defects of *dt* trichomes, we quantitatively measured the geometry of type II trichomes in both WT and *dt* mutants. In the mutants, all trichome cells became shorter and wider, with the most pronounced defect in the first basal cell. The average length of first basal cells in mutants was about 150μm, about one-seventh of that in WT. However, the mean width was about 180μm in mutants which was two times than the WT. The extent of morphological phenotypes in mutant trichomes gradually reduced along the cell file, and the top cell was only half of the WT length while the width was higher than WT (Fig 6F and 6H). Compared to the type II trichomes, the change of overall morphology of type V trichomes was marginal, with cells becoming shorter but much curved (Fig 6D–6E). Unlike type II trichome, the overall morphology of type V was only slightly changed, without visible swollen cells. Furthermore, the phenotype of type III trichomes in *dt* mutants was similar to that observed in type II, with swollen and curved cells (Fig 5Q–5T). Interestingly, the morphological change of glandular trichomes was more like type V non-glandular trichomes, with the stalk cells bending. The glandular cells locating on the top of the type VI trichomes seemed to be WT-like shape and diameter, suggesting the morphology of these cells was independent of the mutant genes (S5 Fig). In addition to trichome cells, the mutations seemed to affect other cell types. The lobe and neck shape of pavement cells of *dt* mutants became substantially decreased (S6 Fig).

### Genes defective in *dt* mutants encode WAVE-ARP2/3 homologs

**Fig 7:**
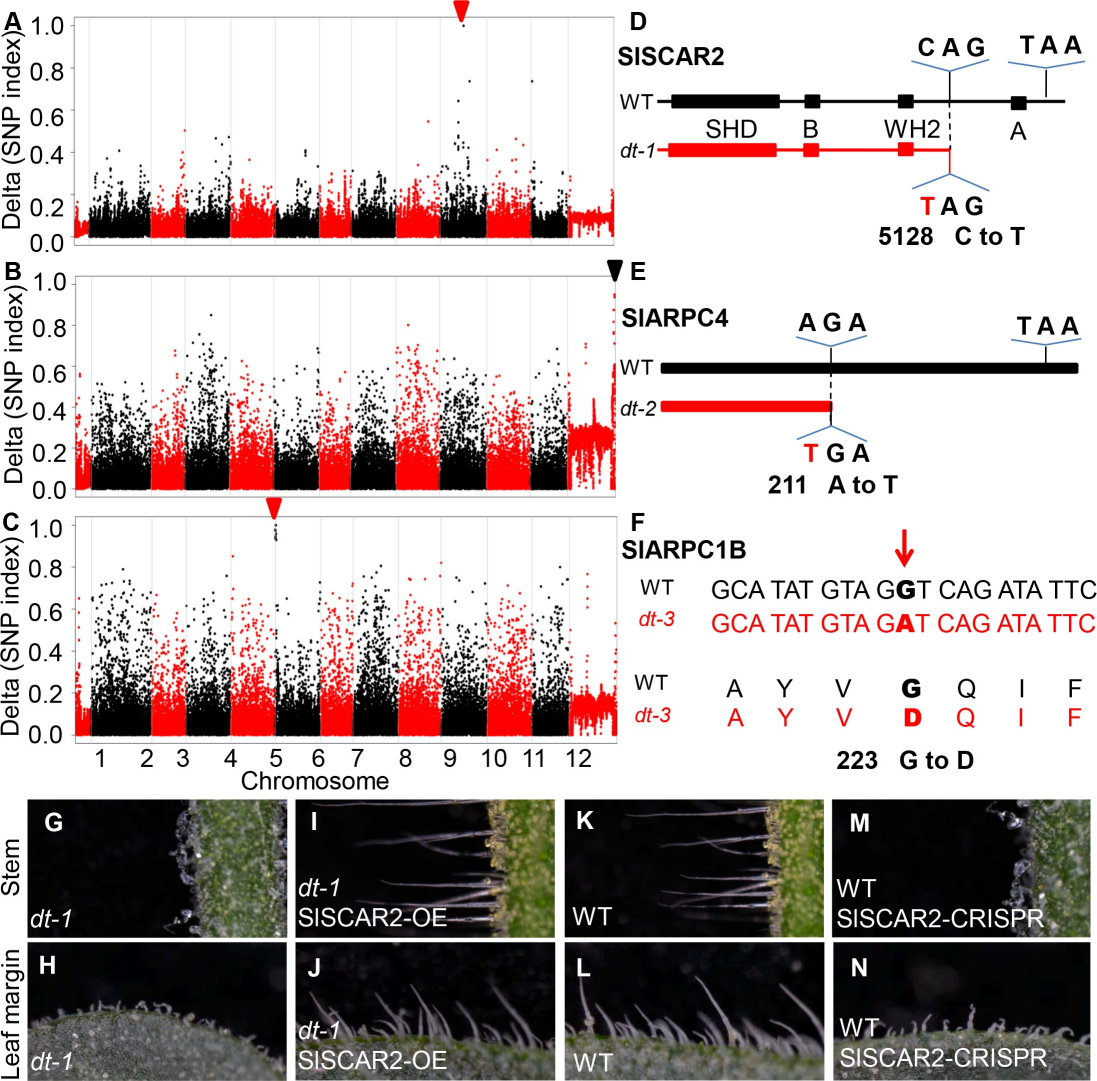
Mapping and verification of the mutation in *dt* mutants. (A-C) Mapping of *dt-1* (A), *dt*-2 (B) and *dt*-3 (C) by BSA-seq analysis. Triangles show the causal mutations with high frequency (Ideally 100% having the variant allele). (D-F) The location of causal mutations in SlSCAR2 of *dt*-1 mutant (D), SlARPC4 of *dt*-2 mutant (E), and SlARPC1B of *dt*-3 mutant (F). (G-N) Complementary verification of SlSCAR2. Over-expression of SlSCAR2 in *dt-2* mutant rescued the distorted trichome phenotype (G-J), and knockout of SlSCAR2 by CRISPR-cas9 in WT resulted in distorted trichomes (K-N).

To identify causative mutations, we developed six F_2_ mapping populations by crossing mutants to the wild type. The trichomes of all F_1_ plants were normal and the separation ratio of F_2_ population was consistent with Mendel's separation law (3:1) (S1 Table). This result indicated that the distorted trichomes phenotype was regulated by recessive genes. Using Next-generation sequencing-based bulked-segregant analysis (BSA) approach, we identified an associated locus on chromosome 9, including two candidate genes, Solyc09g014980 and Solyc09g065130 (Fig 7A). According to the tomato reference genome sequence (https://www.solgenomics.net/), Solyc09g014980 encodes a Wiskott-Aldrich syndrome protein family member and is homology to the *dis3* (AtSCAR2) (Fig 7D). Similar as AtSCAR2, SlSCAR2 consists of four conserved domains, AHD, B, WH2 and A domain [36]. The allele of AtSCAR2 in *dt-1* contained one nucleotide mutation (5128C-T), resulting in an early stop codon and deletion of A domain that is responsible for activating ARP2/3 complex. PCR amplification followed by sequencing of SlSCAR2 from parents and F_1_ population further verified the mutation in the candidate gene (S7 Fig). To further functionally verify the candidate gene, we first performed complementation experiment by stably transforming *dt-1* with the construct carrying the full-length SlSCAR2 driven by CaMV35S promoter. The trichomes on stems and leaves of transgenic plants (28 out of 30 of T0 plants) were rescued by the full-length SlSCAR2 fragment (Fig 7G–7J). Secondly, we knocked out SlSCAR2 in WT by CRISPR/Cas9 and further confirmed the mutation sites by DNA sequencing (S8 Fig). As shown in S4 Fig, we isolated three CRISPR/Cas9 mutant lines. Compared with WT, the trichomes on stems and leaves of all three CRISPR/Cas9 mutants became distorted (Fig 7K–7N).

Using the same strategy, we cloned the mutated genes from other trichome mutants (*dt 1–6*). There are ten candidate genes from BSA analysis of the F2 population of *dt-2* and WT cross. One nucleotide mutation (211A-T) of SlARPC4 resulted in an early stop codon in *dt-2* (Fig 7B and 7E). In *dt-3*, we identified an amino acid substitute (223 G to D) in SlARPC1B. Finally, we identified two candidate genes of ARP2/3 complex and three SCAR/WAVE complex homologs from six *dt* mutants (S2 Table). Two among the five genes we identified (SRA1 and ARPC2A) were also reported previously [33, 37]. As genes identified through our forward genetic screens all belong to the complexes regulating actin filaments, this type of cytoskeletons likely represents an indispensable regulator of trichome morphogenesis.

### The spatial configuration of Actin filaments is altered in *dt* mutants

**Fig 8:**
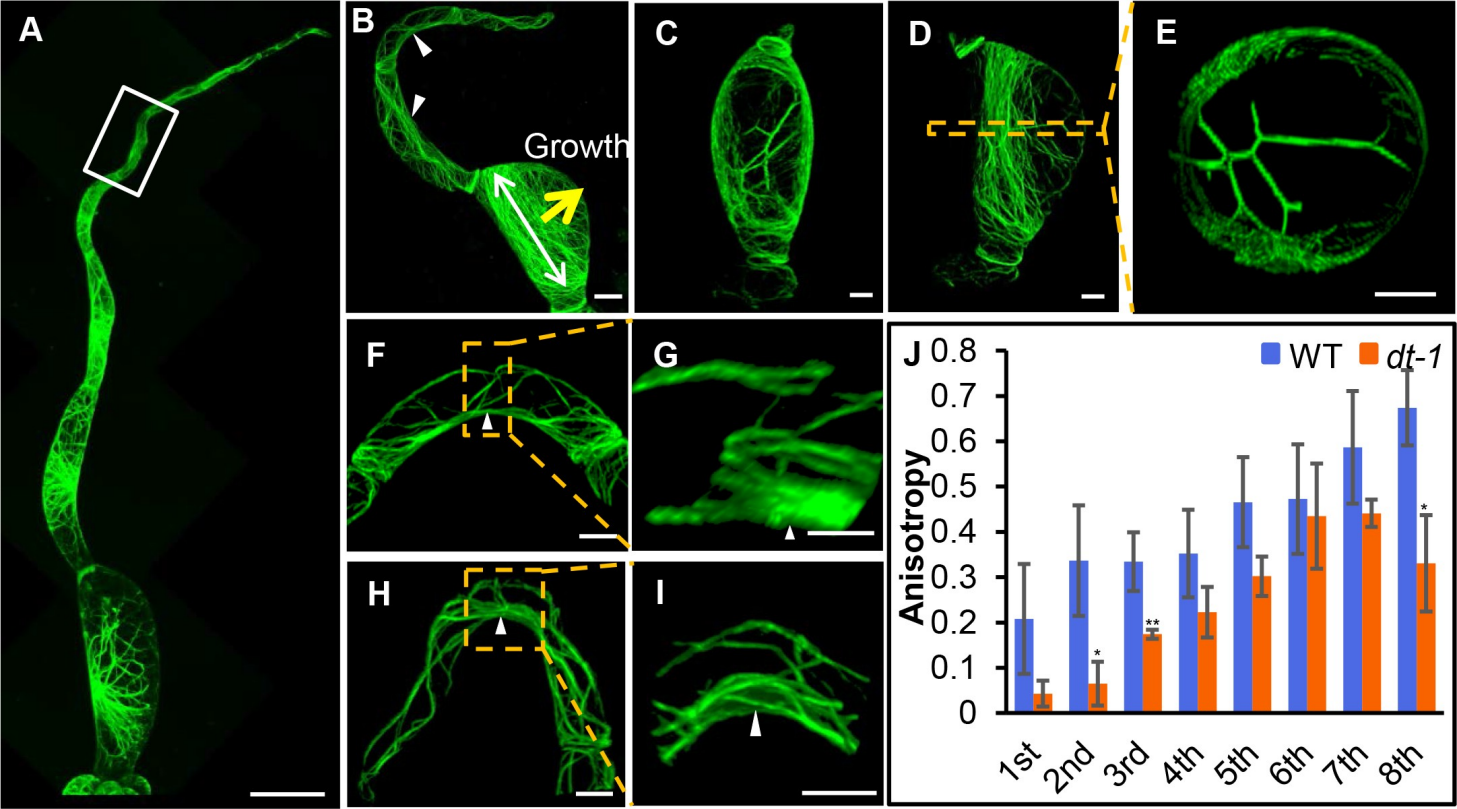
Visualization of actin organization in type II trichomes in *dt-1* mutant. (A) Overall view of actin organization in type II trichomes in *dt-1* mutant. Most of actin filaments orientated randomly. Bar: 20 μm. (B) Overall view of actin organization in type III trichomes in *dt-1* mutant. The alignment of actin filaments derived from the swollen cell. The yellow arrow indicates the major swelling direction; white arrow shows the main orientation of actin filaments. White Triangles point to dense actin bundles. Bar: 20 μm. (C-E) Actin configuration in swollen trichome cells. (E) shows the cross section of the boxed region in (D). Bar: 20 μm. (F, G) Actin organization in the curved cell of the box of (A). Bar: 20 μm. (G) is the amplification of the boxed region in (F). White Triangles point to dense actin bundles. Bar: 10 μm. (H, I) Actin organization in the curved cell locating on the top of type II trichomes. Bar: 20 μm. (I) is the amplification of the boxed region in (H). Triangles point to dense actin bundles. Bar: 10 μm. (J) Quantitative analyses of the anisotropy of actin filaments by FibrilTool. Anisotropy values range from 0 to 1. 0 indicates pure isotropy, and 1 represents pure anisotropy. (*P<0.1 and**P<0.01).

To visualize actin filament organization in *dt* muatnts, we introduced 35S:Lifeact-eGFP into the *dt-1* through stable transformation. Compared with WT, the orientation of actin filament in *dt-1* lost the helix pattern in all trichome cells, and instead formed longitudinal or random actin bundles. Transversely oriented cortical actin meshes in basal and apical region were only observed in some cells (Fig 8A and 8B). In the first basal trichome cell in *dt-1*, the actin often arranged into a girdling belt-like structure that was perpendicular to the growth direction on the most swollen side of the cell. Interestingly, on the other side of the most swollen part, the actin filaments appeared to be diminished (Fig 8B–8D). When visualized in the cross section, the actin appeared to organize not only in the cortical region but also in the cytoplasm, and the amount of cytoplasmic actin cable was significantly increased (Figs 8E and 8J and S9C). In the bending cells, longitudinal actin filaments gathered into cables along the adaxial side of the cell, leading to the aberrantly aggregated actin cables (Figs 8H and 8I and S9A and S9B). Compared with WT, anisotropy of actin filament was significantly reduced in the basal and top cell (Fig 8J), which was consistent with the severe cell morphology.

### Microtubules are essential for actin organization in tomato trichomes

**Fig 9:**
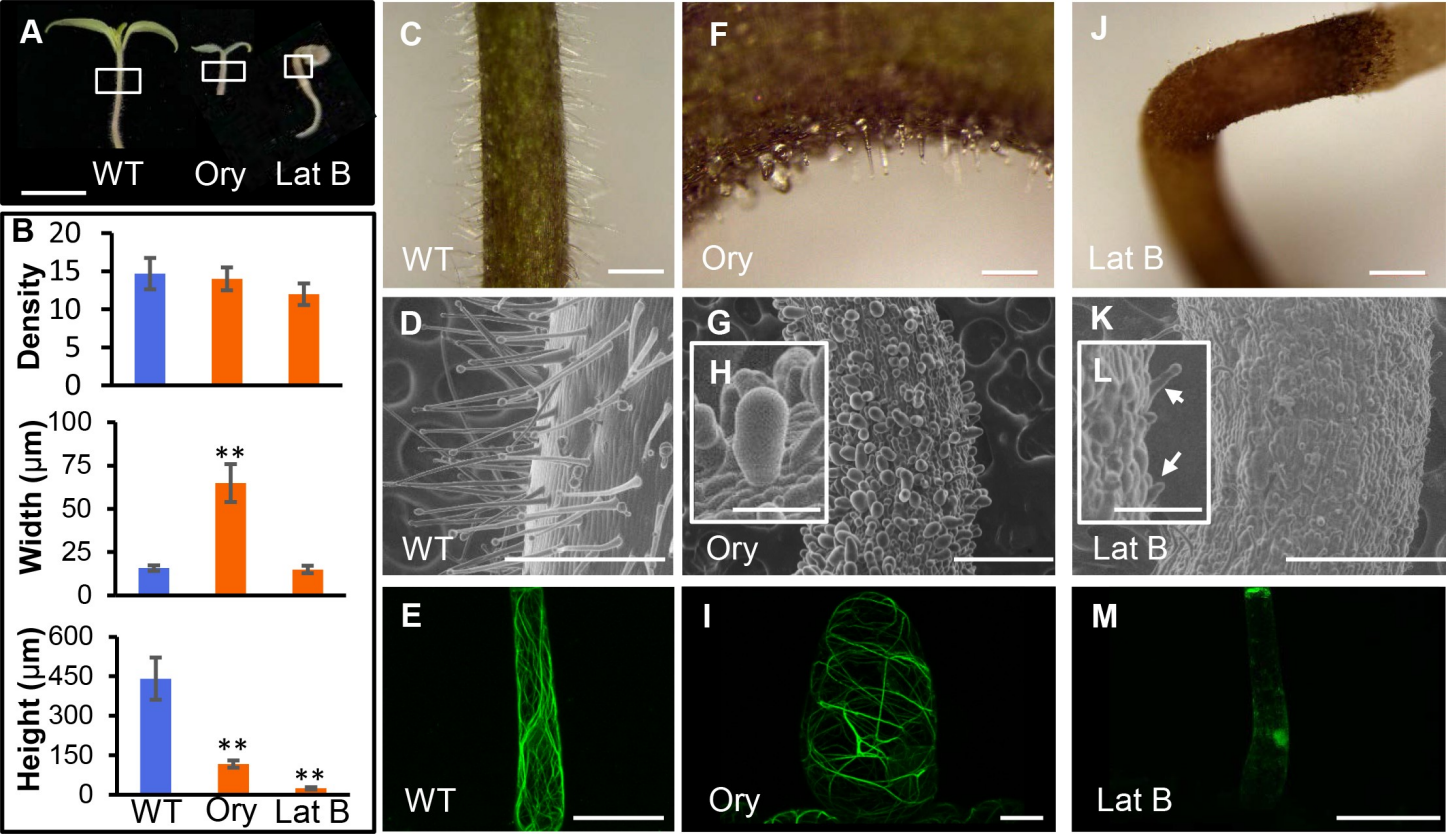
Effect of pharmacological disruption of microtubules and actin filaments on trichomes. (A) The seedling phenotype after four-day treatment with Oryzalin (Ory) and Latrunculin B (Lat B). (B) Disruption of microtubules and actin filaments dramatically changed the trichome morphology but did not inhibit trichome initiation (Statistic analysis of the trichomes density of over 100 hundred epidermal cells for each treatment). (C-E) Trichome morphology and Lifeact-eGFP in WT trichomes. (F-I) Treatment with Ory resulted in isotropically expanded cells (G, H) and horizontal actin bundles (I). (J-M) Treatment with Lat B caused the entire depolymerization of actin filaments (M) and decreased cell elongation (K, L). (D, G, J) are SEM micrographs and (E H K) are confocal images. A, Bar: 1 cm. C and I, Bar: 500 μm. F, Bar: 250 μm. D, G and J, Bar: 500 μm. E, H and K, Bar: 50 μm. H and L, Bar: 100 μm.

To functionally dissect the roles of microtubules and actin filaments in tomato trichomes, we treated the tomato seedlings with 20μm/L Oryzalin and 20μm/L Latrunculin B for four days respectively (Fig 9A). Both disrupted microtubules and actin filaments remarkably inhibited trichome morphology (Fig 9A, 9C, 9F and 9J). With Oryzalin treatment, all trichome cells became radially swollen, resembling the isotropic expansion of microtubule-disrupted root cells (Fig 9G and 9H). When actin configuration was abolished, the axial extension of trichome cells was significantly repressed while the overall anisotropy seemed to be retained (Fig 9K and 9L). Interestingly, Oryzalin treated cells with swollen shape exhibited increased bundling of actin cables and reduced fine actin filaments (Figs 9I and S9D). In addition, the thick actin cables appeared to align more transversely, possibly girdling the expansion force. These results suggest that microtubules presumably function as a major player to maintain the cell expansion anisotropy while actin filaments are more important to promote the axial elongation of the cell in trichomes. We further quantified the trichome initiation using SEM images. Despite actin filaments were all removed by the treatment, trichome initiation was only slightly affected. Similarly, disassembly of microtubules seemed to only influence the anisotropy of cell expansion, without affecting the trichome initiation rate (Fig 9B). Thus cytoskeletons possibly control tomato trichomes mainly through morphological regulation.

## Discussion

**Fig 10:**
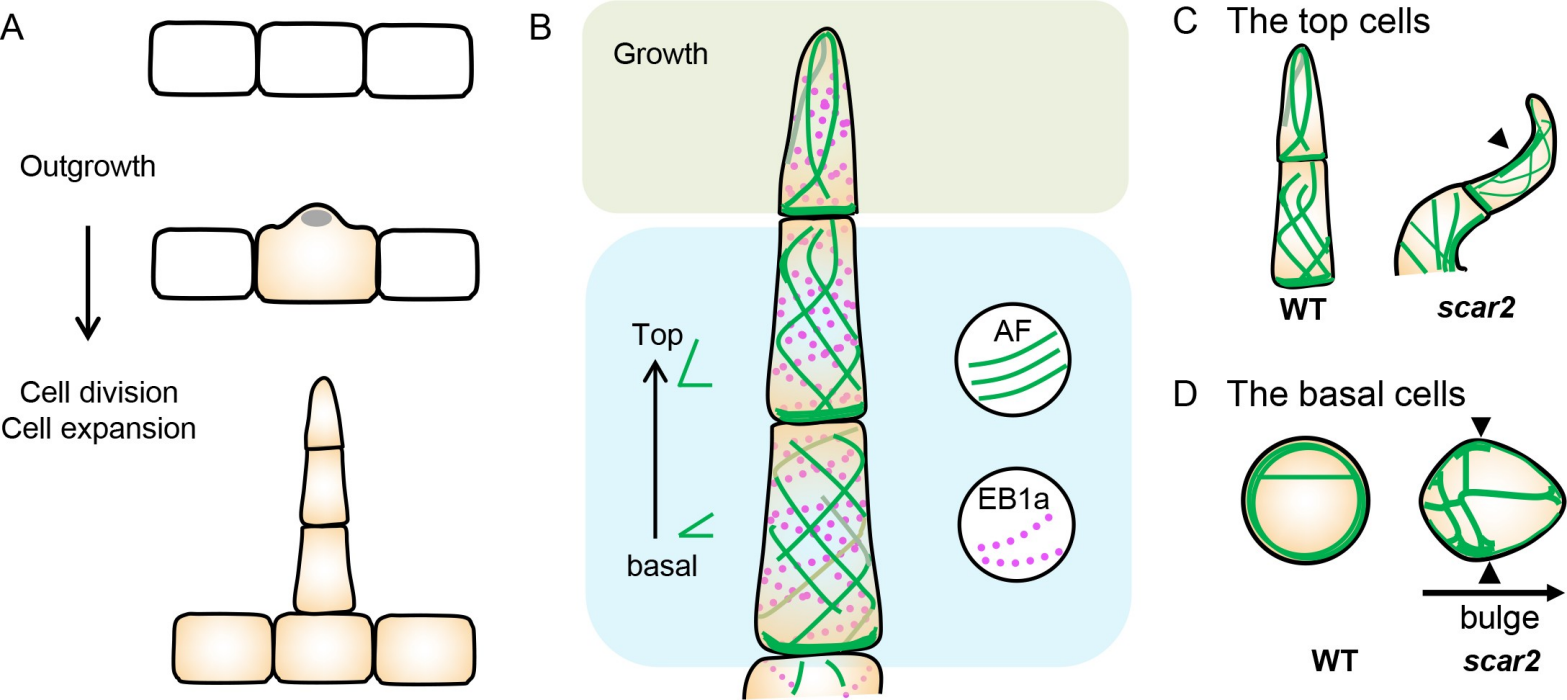
Schematic illustration of the role of actin filaments (AFs) and micro tubules (MTs) in multicellular trichome development. (A) The morphogenesis of the multicellular trichomes. The first step is the outgrowth of the cell. At this stage, the nucleus enters into the bulging site. The second step is the cell division and cell expansion. The grey oval shows the nucleus. (B) The organization of Liftact decorated AFs and EB1 decorated MTs during trichome cell morphogenesis. The green lines show the organization of Liftact decorated AFs. The red dots show the signal of EB1a. In the apical cell, the orientation of actin filaments is longitudinal which may promote the polar growth of the cell. In the middle and basal cells, actin filaments are spirally organized. The green angles mean that the alignment of AFs gradually changes from transverse and spiral to longitudinal in cells from the basal to the top. (C D) The organization of actin filaments in WT and *dt-2* mutant. The mutation of SCAR2 leads to misshaped cells. (C) In the top bent cells, actin cables align longitudinally in WT while gathered on adaxial side of the cell (pointed by black triangle) in *scar*. (D) In the swollen cell in *scar-2*, aggregated actin bundles were often observed in the cortical region of the cell (black triangle), and thick actin cables in the cytoplasm was more frequent than WT.

Both microtubules and actin filaments have been shown to promote morphogenesis of unicellular trichome in Arabidopsis. Previous cell imaging and genetics evidence suggest that microtubules are more involved in trichome branching in Arabidopsis [29–31]. However, the regulation in multicellular tomato trichomes appeared to be more complex. Microtubules seemed to control expansion anisotropy which is similar to its canonical roles in many other cell types [38]. The tomato trichome initiated with the nuclear moving into the bulging site, and both microtubules and actin filaments formed longitudinal network to promote the protrusion from epidermis (Fig 10A). The spiral pattern and gradual transition from transverse to longitudinal actin alignment presumably represent the different stage of trichome expansion. Furthermore, the cell length of the top cell is more variable than the basal cell. The basal cells could reflect the more fully expanded status while the rest cells are still in expanding. Since most cells within a trichome need to increase both length and width, it is reasonable to infer that most trichome cells undergo diffuse expansion. However, compared to the canonical cytoskeleton organization in diffusely expanding cells, both actin filaments and microtubules appear to organize in dissimilar manner in different trichome cells in tomato. During expansion, root cells form transverse cortical microtubules and longitudinal actin filaments while leaf pavement cells have actin aggregated in the lobe protruding region and microtubules bundled at the indentation [39]. In tomato trichomes, transverse alignment of both actin and microtubules are restricted to fully expanded basal cells or two ends of an expanding cell. In the middle part between the apical and basal end of a trichome cell, both cytoskeletons form spiral organization which could be important for promoting the cone-shape. In addition, actin filaments organize in the similar helix as microtubules, suggesting a potential coordination between the two cytoskeletons (Fig 10B). When microtubule was disrupted by oryzalin, actin filaments bundled into cables and lost longitudinal alignment in trichome cells, providing further support of the interaction between the two cytoskeletons.

Although the paradigm of cytoskeletons in cell expansion is directing the arrangement of cell wall components, actin filaments in trichomes also seemed to respond to mechanics derived from cell expansion [13, 40, 41]. The spiral pattern of actin filaments in most cells between the basal one and the distal top one presumably results from the reciprocal interaction between cytoskeletons and cell wall. The spatial distribution of cytoskeletons controlled by mechanical heterogeneity within a cell was previously reported in a number of cellular systems [5, 42] However, the correlation between cytoskeletons and mechanical force seems to be dissimilar in different systems. In epidermal pavement cells, the microtubules were often found to organize in bundles parallel to the direction of maximal tensile stress [3, 43]. During trichome branching in Arabidopsis, microtubules reorient circumferentially in the collar region of the emerging site [29]. In tomato trichomes, both microtubules and actin filaments gradually re-orient from transverse array to helical organization, and eventually to longitudinal alignment in top cells. This pattern possibly reflects the mechanical distinction derived from cell expansion in those cells. Besides, the changing direction of cytoskeletons in different cells could associate to different expansion stages of trichome cells.

In addition, the actin filaments in *dt* mutants re-aligned into the girdling structure along the maximal mechanical forces caused by swollen trichome cells. Intriguingly, we observed a large number of actin bundles formed across the cytoplasm connecting expanding cell walls on different sides (Fig 10C). These observations provide indirect evidence supporting actin filaments interplay with mechanical stress within a cell. During expansion, the cell wall exhibits a great extent of elasticity through constantly loosening and stiffening. Thus the possible scenario is cytoskeletons not only provide a mechanical constraint to the cell wall elasticity, but alter their own alignments in response to the tensile stress, giving a feedback to amplify the outgrowth signal and eventually shape the cell.

It was observed in Arabidopsis trichome that the early bulging epidermis has few cortical microtubules while contains prominent actin meshwork [44]. By contrast, microtubules seemed to play a major role in trichome branching [30, 31]. The apical zone of the trichome branching always contains a microtubule-depleted zone while microtubules form transverse bundles at the base of protruding tip [28]. In contrast, live-imaging of EB1a marker showed that there was EB1a-GFP signal in the apex of the top trichome cells (Fig 4B and 4F). However, whether there is a microtubule configuration in protruding domain of tomato trichomes remains to be further verified using more direct markers including GFP-Tubulin or GFP-MBD. We also observed longitudinal cytoskeleton bundles of bulging site of epidermis, which suggest that actin filament and microtubule cytoskeletons may play distinct roles in polar growth of the tomato trichome. In the elongating tomato trichome cells, both cytoskeletons were either longitudinally or spirally aligned along the growth axis, with the transverse organization visualized only in basal cells or apical and basal ends of other elongating cells. However, this pattern is not specific to multicellular structures as it was recently observed that unicellular cotton fiber also has a longitudinal actin filament with transversely aligned microtubules [32]. When tomato trichomes were treated by oryzalin, all trichomes became isotropically expanded balloon like structure, indicating microtubules play canonical roles in cell expansion. By contrast, disruption of actin filaments by latrunculin B produced short trichomes with normal anisotropy, suggesting actin filaments are involved more in cell elongation. Interestingly, pharmacologically disrupted either cytoskeletons failed to inhibit the trichome outgrowth from epidermis. In addition, we observed the nuclear movement into the bulging site before the significant outgrowth in epidermis, suggesting the bulging process is likely regulated by a cytoskeleton independent mechanism.

The major role of actin in trichome cell elongation can be further supported by the observation of predominant arrangement of actin along the extension axis in the top cells, resembling the actin structures in tip-growing cells. In tip growth, actin bundles, rather than microtubules function to direct the deposition of cell wall materials via secretion [45]. The actin cables determine the location and direction of cell growth by targeted secretion of vesicles containing cell wall materials transporting along actin filament. In addition, actin cables could also provide the mechanical force for the outgrowth as suggested in pavement cell lobes and trichome initiation in Arabidopsis. The longitudinal alignment of actin in top cells of trichomes could drive the directional vesicle transport and provide leading force for the cell elongation. The location and direction of the cell protrusion seemed to reply mainly on proper actin configuration as loss of SCAR/WAVE and ARP2/3 complex led to substantially curving cells. In *dt* mutants in which actin filaments became defective and bundled, most cells except for the basal one became distorted and curved but maintained the overall expansion anisotropy. Together with the pharmacological assay of actin filaments in trichome morphology, our observations suggest that microtubules and actin function on different aspects of the trichome cell morphology with microtubules more on expansion anisotropy and actin more on trichome elongation.

## Materials and methods

### Plant materials, mapping populations and growth conditions

Trichomes mutants were isolated from an EMS mutant tomato in miniature cultivar Micro-Tom (TOMJPF00001). All seeds of WT and mutant lines were provided by TOMATOMA (http://tomatoma.nbrp.jp/) (Saito et al., 2011). Trichomes mutants were screened form the following accession numbers: *dt-1* (TOMJPW3695-1), *dt-2* (TOMJPE7376-1), *dt-3* (TOMJPW4375), *dt-4* (TOMJPW2352-1), *dt-5* (TOMJPE3936-2), *dt-6* (TOMJPW2193). All parents, F2 populations and transgenic plants were grown in the greenhouse with a 16h light/8h dark cycle, 18°C to 30°C temperature.

### Morphological and cellular analysis

The abundance analysis of trichomes was conducted on the stem between third and fourth leaves of 30 days seedlings using stereomicroscope (Nikon, SMZ18). More than 6 independent WT plants and 4 independent mutant plants were measured. For quantification of the geometric parameters of trichomes, images of trichomes was taken by DIC (Nikon, ECLIPSE Ni-U) and cell number of each trichomes, cell length and cell width was manually measured using Image J software. More than 200 trichomes from 10 independent WT plants and 30 trichomes from 6 independent mutant plants were measured.

### Construction of marker lines

The Lifeact, a 17-amino-acid peptide that is derived from the yeast actin binding protein ABP-140 [46], is regarded as being the most authentic marker for actin organization and distribution. Microtubule end-Binding protein EB1 binds to all growing MT plus-ends. Lifeact and EB1a (At3g47690), fused with eGFP respectively, were introduced into the pHellsgate 8 vector under control of the CaMV 35S promoter. The construction of 35S::Lifeact-eGFP and 35S::EB1-eGFP were transformed into Micro-Tom tomatoes by Agrobacterium-mediated genetic transformation.

### Quantification methods

For the quantification of the geometric parameters of trichome cells, cell heights, cell widths and actin angles were manually measured using ImageJ software. More than 200 trichome cells from 10 independent wild-type plants and 30 trichome cells from 6 independent mutant plants were measured. FibrilTool, an ImageJ plug-in, was used for quantification of the orientation angle and the anisotropy of actin arrays in a given region of interest of the wild type and *dt*-1. Anisotropy values range from 0 to 1. 0 indicates pure isotropy, and 1 represents pure anisotropy. Statistical analyses were performed using standard’s t test. Data were represented as the mean ± SE from at least three independent experiments. Not significant P > 0.05, 0.01 <*P < 0.05, **P < 0.01.

### Confocal laser scanning microscopy

To visualize actin and microtubules, trichomes of transgenic plants stably expressing Lifeact-eGFP or EB1-eGFP were imaged by a Zeiss LSM 880 confocal laser scanning microscope with a 40× water lens. For imaging 3D reconstruction of trichomes, Z stacks of confocal images from the distal regions of trichomes with stably expressing green fluorescence were taken from the top view along the Z axis at steps of 0.8 μm, and were used to reconstruct the 3D images using Zeiss LSM 880 software.

### Mapping populations

Mapping populations were constructed by crossing WT (Micro-Tom, female) and trichomes mutant (male). F2 population obtained by selfing the F1. To determine the mutation is whether mono-gene recessive or not, F2 segregation analysis was conducted.

### Bulked segregant analysis (BSA)

Genomic DNA of 30 individuals clearly exhibiting mutant-like phenotypes from F_2_ population and 30 WT individuals was extracted by CTAB. All DNA quality and concentration were checked and then mixed to construct two bulks (mutant-like bulk; WT bulk). Each bulk was sequenced to a depth of 30× coverage of the tomato genome by HiseqXten-PE150 (Novogene). Trimmed sequences are mapped onto the tomato reference genome (Heinz 1706 cultivar) and EMS mutation variants are filtered. Analysis of the allelic variant frequencies in the pools leads to the identification of the causal mutation with very high frequency in the mutant-like bulk (ideally 100% having the variant allele). The genes with the expected allelic frequency tend to be 1 for the mutant-like bulk was performed mutation identification and transgenic verification [47]. Then, the candidate genes were cloned and sequenced to verify the mutant site. The primers were listed in the S3 Table.

### Transgenic analysis

To verify the candidate genes, two expression vectors were constructed. The full-length coding sequence of candidate genes from WT was inserted into the pHellsgate 8 vector under control of the CaMV 35S promoter. For construction of CRISPR vector, target sequence was designed based on CRISPR-PLANT (https://www.genome.arizona.edu/crispr/). Then, the target sequences were introduced into the pTX vector in which the target sequence was driven by the tomato U6 promoter and Cas9 by 2×35S. The recombinant pHellsgate8 vectors were transformed into corresponding mutants and CRISPR vectors were transformed into WT, mediated by Agrobacterium tumefaciens strain C58. The primers were listed in the S3 Table.

### Latrunculin B (Lat B) and Oryzalin (Ory) treatment

Freshly sprouted tomato seeds were treated on the 1/2 MS medium with 20mmol/L Lat B and 20mmol/L Ory for four days. Controls and treatments were grown in the growth chamber with a 16h light/8h dark cycle, 22°C temperature. Phenotypes were imaged using DIC (Nikon, ECLIPSE Ni-U), scanning electron microscopic (SEM) and Zeiss LSM 880 confocal laser scanning microscope.

### Antibodies and Immuno-staining

For immunostaining, trichomes with sliced leaves were fixed for 1h at room temperature with 4% paraformaldehyde in 50 mM PIPES, pH 6.8, 5 mM EGTA, pH7.0, 2 mM MgCl_2_, and 0.4% Triton X-100. The fixative was washed away with PBS buffer, and samples were treated for 15 mins at room temperature with the solution of 0.1% Cellulase R-10 and 0.1% Pectinase Y-23. And then, samples were incubated for 30 min in 1% (w/v) BSA in PBS and incubated for 1 h with monoclonal anti-α-tubulin-fluorescein isothiocyanate antibody produced in mouse (1:2000, Sigma F2168) at 37°C. Samples were then washed three times for 10 mins in PBS and incubated for 1 h with secondary antibodies (1:500) at 37°C. After washing in the PBS buffer with 0.1% p-Phenylenediamine, 0.1 M Propyl gallate, 50% Glycerol, pH9.5, samples were imaged using the Zeiss LSM880 confocal microscopy.

## Supporting information

S1 Fig The morphology of type II and V trichomes. (A, B) The abundance of each trichome type.
(C) Schematic diagram of the type II trichome. The basal cell that directly connects to the leaf epidermis is the first cell and the distal top cell is the eighth cell.
(D, E) Cell length and cell width of each cell within type II trichome cell file.
(F) Schematic diagram of the type V trichome.
(G, H) Cell length and cell width of each cell within type V trichome cell file.
(I) Scatter diagram of cell length of different cells within type II trichome cell file. Y-axis shows the number of trichomes used for measuring cell length. X-axis shows the cell length of different cell (μm).
(TIF)

S2 Fig The morphology of transgenic plants expressing 35S: Lifeact-eGFP. (A B) WT (A) and the normal transgenic plants (B). Bar:10 cm.
(C D) The normal and abnormal actin alignment in transgenic plants with abnormal morphology. Bar:25 μm.
(E F) The normal actin alignment in the head of the type VI trichome (E) and the top cell of type II trichom (E) in the normal transgenic plants. Bar:20 μm.
(G H) Immuno-staining images of actin filaments in the top cell (G) and the stalk cell of trichomes (H) in WT using anti-actin. Bar: 25 μm.
(TIF)

S3 Fig Visualization of actin organization in type V trichomes using Lifeact-eGFP fusion protein. (A) A panoramic micrograph of actin organization in the type V trichome cell file. Bar: 50 μm.
(B-D) Details of actin arrangement in each cell of the type V trichomes.
E) Average orientation of cortical actin filaments in type V trichomes by Image J.
(TIF)

S4 Fig The morphology of transgenic plants expressing 35S: EB1-eGFP. (A B) WT (A) and the transgenic plants (B); Bar: 10cm.
(C D) The signal of EB1a-GFP in type V trichomes (C) and stomata (D). Bar: 20 μm.
(E-G) Immuno-staining image of microtubules in the stomata (E), the mesophyll cells (F) and the stalk cell of trichomes (G) in WT using anti-tubulin. Bar:20 μm.
(TIF)

S5 Fig Morphological comparison of the type VI glandular trichomes between WT and *dt* mutants. (A B) Phenotype of type VI glandular trichomes of WT and *dt* mutants by SEM. Bar: 100 μm.
(C) Diameter of the gland heads of type VI trichomes.
(TIF)

S6 Fig *dt* mutants affect the morphology of pavement cells. (A-D) SEM micrographs showing pavement cell shape in the WT (A) and *dt* mutants.
(C-D). The lob and neck were shown in the (A). Bar: 200 μm.
(E) Average width of the neck in the WT and *dt* mutants. (*P<0.1 and**P<0.01). (F) Average length of the lob in the WT and *dt* mutants. (*P<0.1 and**P<0.01)
(TIF)

S7 Fig Verification of the mutation of SCAR2, ARPC4 and ARPC1 in WT, *dt* mutants and F1 plants. (TIF)

S8 Fig CR-scar2 alleles identified from three T1 mutant lines. Allele sequences that were determined by sequencing are shown.
(TIF)

S9 Fig The quantification of F-actin filaments (AFs) and actin bundles. (A) The quantification of cortical actin filaments (AFs) in the basal cell (BC) and the top cell (TC) in the WT and *dt-2*.
(B) The quantification of cortical actin cables in the basal cell (BC) and the top cell (TC) in the WT and *dt-2*.
(C) The quantification of actin cables in the cytoplasm of the basal cell.
(D) The quantification of the F-actin filaments and cables after Oryzalin treatment.
(TIF)

S1 Table Genetic analysis in different F2 populations. (XLSX)

S2 Table Genes encoding subunits of the ARP2/3 and WAVE complexes in tomato and Arabidopsis. (XLSX)

S3 Table PCR primers used in this paper. (XLSX)

S1 Video The F-actin dynamics at the trichome initiation stage. Related to Fig 3A–3F. Bar: 8 μm. The parameter is 28 *z*-slices, 1 μm step size and 13 time series.
(MP4)

S2 Video The F-actin dynamics at the one-cell stage. Related to Fig 3G–3I. Bar: 5 μm. The parameter is 19 *z*-slices, 1 μm step size; 60 time series.
(MP4)

S3 Video The F-actin dynamics at the four-cell stage. Related to Fig 3K–3P. Bar: 50 μm. The parameter is 58 *z*-slices, 1 μm step size; 20 time series.
(MP4)

S4 Video The EB1a-GFP dynamics at the two-cell stage. Related to Fig 4I and 4J. Bar: 5 μm. The parameter is 10 *z*-slices, 1 μm step size; 20 time series.
(MP4)

S5 Video The EB1a-GFP dynamics at the three-cell stage. Related to Fig 4K and 4L. Bar: 5 μm. The parameter is 10 *z*-slices, 1 μm step size; 20 time series.
(MP4)

## References

[1] Powell AE, Lenhard M. Control of organ size in plants. Curr Biol, 2012, 22: R360–R367. 10.1016/j.cub.2012.02.010 22575478

[2] Tsukaya M. Mechanism of leaf-shape determination. Annu Rev Plant Biol, 2006, 57: 477–496. 10.1146/annurev.arplant.57.032905.105320 16669771

[3] Fu Y, Gu Y, Zheng Z, Wasteneys G, Yang, Z. Arabidopsis interdigitating cell growth requires two antagonistic pathways with opposing action on cell morphogenesis. Cell, 2005, 120: 687–700. 10.1016/j.cell.2004.12.026 15766531

[4] Scheres B, Wolkenfelt H. The Arabidopsis, root as a model to study plant development. Plant Physiol Bioch, 1998, 36:21–32.

[5] Sampathkumar A, Yan A, Krupinski P, Meyerowitz EM. Physical forces regulate plant development and morphogenesis. Curr Biol, 2014, 24: R475–R483. 10.1016/j.cub.2014.03.014 24845680 PMC4049271

[6] Sambade A, Findlay K, Schaffner AR, Meyerowitz E. Actin dependent and -independent functions of cortical microtubules in the differentiation of Arabidopsis leaf trichomes. Plant Cell, 2014, 26, 1629–1644. 10.1105/tpc.113.118273 24714762 PMC4036576

[7] Luckwill L.C. The genus Lycopersicon: A historical, biological and taxonomic survey of the wild and cultivated tomato. Aberd Univ Stud, 1943, 120:1–44.

[8] Fu Y. The cytoskeleton in the pollen tube. Curr Opin Plant Biol, 2015, 28:111–119. 10.1016/j.pbi.2015.10.004 26550939

[9] Li J, Wang X, Qin T, Zhang Y, Liu X, Sun J, et al MDP25, a novel calcium regulatory protein, mediates hypocotyl cell elongation by destabilizing cortical microtubules in Arabidopsis. Plant Cell, 2011, 23:4411–4427. 10.1105/tpc.111.092684 22209764 PMC3269874

[10] Galatis B, Apostolakos P. The Role of the cytoskeleton in the morphogenesis and function of stomatal complexes. New Phytol, 2010, 161:613–639. 10.1046/j.1469-8137.2003.00986.x 33873710

[11] Ren H, Dang X, Cai X, Yu P, Li Y, Zhang S, et al Spatio-temporal orientation of microtubules controls conical cell shape in Arabidopsis thaliana petals. Plos Genet, 2017, 13(6): e1006851 10.1371/journal.pgen.1006851 28644898 PMC5507347

[12] Van BN, Joss G, Van OP. Reorganization and in vivo dynamics of microtubules during Arabidopsis root hair development. Plant Physiol, 2004, 136(4):3905–3919. 10.1104/pp.103.031591 15557102 PMC535824

[13] Baskin TI, Beemster GT, Judy-March JE, Marga F. Disorganization of cortical microtubules stimulates tangential expansion and reduces the uniformity of cellulose microfibril alignment among cells in the root of Arabidopsis. Plant Physiol, 2004, 135:2279–2290. 10.1104/pp.104.040493 15299138 PMC520797

[14] Hepler PK, Vidali L, Cheung AY. Polarized cell growth in higher plants. Annu Rev Cell Dev Bi, 2001, 17:159–187. 10.1146/annurev.cellbio.17.1.159 11687487

[15] Menand B, Calder G, Dolan L. Both chloronemal and caulonemal cells expand by tip growth in the moss Physcomitrella patens. J Exp Bot, 2007, 58:1843–1849. 10.1093/jxb/erm047 17404383

[16] Smith LG, Oppenheimer DG. Spatial control of cell expansion by the plant cytoskeleton. Annu rev cell Dev Bi. 2017, 21, 271–95. 10.1146/annurev.cellbio.21.122303.114901 16212496

[17] Sheahan M. B, Staiger, CJ, Rose RJ, McCurdy DW. A green fluorescent protein fusion to actin-binding domain 2 of Arabidopsis fimbrin highlights new features of a dynamic actin cytoskeleton in live plant cells. Plant Physiol, 2004, 136: 3968–3978. 10.1104/pp.104.049411 15557099 PMC535829

[18] Le J, El-Assal SE, Basu D, Saad ME, Szymanski DB. Requirements for Arabidopsis ATARP2 and ATARP3 during epidermal development. Curr Biol, 2003, 13: 1341–1347 10.1016/s0960-9822(03)00493-7 12906796

[19] Kost B, Spielhofer P, Chua NH. A GFP-mouse talin fusion protein labels plant actin filaments in vivo and visualizes the actin cytoskeleton in growing pollen tubes. Plant J, 1998, 16: 393–401 10.1046/j.1365-313x.1998.00304.x 9881160

[20] Vidali L, Rounds CM, Hepler PK, Bezanilla M. Lifeact-mEGFP reveals a dynamic apical F-actin network in tip growing plant cells. Plos One, 2009, 4: e5744 10.1371/journal.pone.0005744 19478943 PMC2684639

[21] Zhang M, Zhang R, Qu X, Huang S. Arabidopsis FIM5 decorates apical actin filaments and regulates their organization in the pollen tube. J Exp Bot, 2016, 67(11):3407–3417. 10.1093/jxb/erw160 27117336 PMC4892729

[22] Lovy-Wheeler A, Kunkel JG, Allwood EG, Hussey PJ, Hepler PK. Oscillatory increases in alkalinity anticipate growth and may regulate actin dynamics in pollen tubes of lily. Plant Cell, 2005, 18, 2182–2193. 10.1105/tpc.106.044867 PMC1560910 16920777

[23] Gu Y, Fu Y, Dowd P, Li S, Vernoud V, Gilroy S et al A Rho family GTPase controls actin dynamics and tip growth via two counteracting downstream pathways in pollen tubes. J Cell Biol, 2005, 169(1):127–138. 10.1083/jcb.200409140 15824136 PMC2171904

[24] Qu X, Zhang H, Xie Y, Wang J, Chen N, Huang S. Arabidopsis Villins promote actin turnover at pollen tube tips and facilitate the construction of actin collars. Plant Cell, 2013, 25: 1803–1817. 10.1105/tpc.113.110940 23715472 PMC3694707

[25] Qu X, Zhang R, Zhang M, Diao M, Xue Y, Huang S. Organizational innovation of apical actin filaments drives rapid pollen tube growth and turning. Mol Plant, 2017(7): 930–947. 10.1016/j.molp.2017.05.002 28502709

[26] Basu D, Le J, El-Essal Sel D, Huang S, Zhang C, Mallery EL, et al DISTORTED3/SCAR2 is a putative Arabidopsis WAVE complex subunit that activates the Arp2/3 complex and is required for epidermal morphogenesis. Plant Cell, 2005, 17:502–24. 10.1105/tpc.104.027987 15659634 PMC548822

[27] Mathur J, Mathur N, Kernebeck B, Hülskamp M. Mutations in actin-related proteins 2 and 3 affect cell shape development in Arabidopsis. Plant Cell, 2003, 15:1632–45. 10.1105/tpc.011676 12837952 PMC165406

[28] Yanagisawa M, Desyatova AS, Belteton SA, Mallery EL, Turner JA, Szymanski DB. Patterning mechanisms of cytoskeletal and cell wall systems during leaf trichome morphogenesis. Nature Plants, 2015, 1(3):15014. 27246881 10.1038/nplants.2015.14

[29] Folkers U, Kirik V, Schöbinger U, Falk S, Krishnakumar S, Pollock MA, et al The cell morphogenesis gene ANGUSTIFOLIA encodes a CtBP/BARS-like protein and is involved in the control of the microtubule cytoskeleton. EMBO J, 2002, 21:1280–8. 10.1093/emboj/21.6.1280 11889034 PMC125931

[30] Oppenheimer DG, Pollock MA, Vacik J, Ericson B, Feldmann K, Marks MD. Essential role of a kinesin-like protein in Arabidopsis trichome morphogenesis. P Natl Acad Sci, 1997, 94:6261–6. 10.1073/pnas.94.12.6261 PMC21037 9177205

[31] Torres-Ruiz RA, Jurgens G. Mutations in the FASS gene uncouple pattern formation and morphogenesis in Arabidopsis development. Development, 1994, 120:2967–78. 10484674 10.1242/dev.120.10.2967

[32] Yu Y, Wu S. Nowak J, Wang G, Han L, Feng Z, et al Live-cell imaging of the cytoskeleton in elongating cotton fibres. Nat plants, 2019, 5, 498–504. 10.1038/s41477-019-0418-8 31040442

[33] Kang JH, Campos ML, Zemelis-Durfee S, Al-Haddad JM, Jones AD, Telewski FW, et al Molecular cloning of the tomato *Hairless* gene implicates actin dynamics in trichome-mediated defense and mechanical properties of stem tissue. J Exp Bot, 2016, 67(18):5313–5324. 10.1093/jxb/erw292 27481446 PMC5049383

[34] Szymanski DB. Breaking the WAVE complex: the point of Arabidopsis trichomes. Curr Opin Plant Biol, 2005, 8(1):103–112. 10.1016/j.pbi.2004.11.004 15653407

[35] Mathur J, Chua NH. Microtubule stabilization leads to growth reorientation in Arabidopsis trichomes. Plant Cell, 2000, 12, 465–477. 10.1105/tpc.12.4.465 10760237 PMC139846

[36] Zhang C, Mallery EL, Schlueter J, Huang S, Fan Y, Brankle S, et al Arabidopsis SCARs function interchangeably to meet actin-related protein 2/3 activation thresholds during Morphogenesis. Plant Cell, 2008, 20:995 10.1105/tpc.107.055350 18424615 PMC2390748

[37] Jeong NR, Kim H, Hwang IT, Howe GA, Kang JH. Genetic analysis of the tomato inquieta mutant links the ARP2/3 complex to trichome development. J Plant Biol, 2017, 60(6):582–592.

[38] Ren H, Dang X, Yang Y, Huang D, Liu M, Gao X, et al SPIKE1 Activates ROP GTPase to modulate petal growth and shape. Plant Physiol, 2016, 172:358–371. 10.1104/pp.16.00788 27440754 PMC5074625

[39] Fu Y, Li H, Yang Z. The ROP2 GTPase controls the formation of cortical fine F-actin and the early phase of directional cell expansion during Arabidopsis organogenesis. Plant Cell, 2002, 14:777–94. 10.1105/tpc.001537 11971134 PMC150681

[40] Paredez AR, Somerville CR, Ehrhardt DW. Visualization of cellulose synthase demonstrates functional association with microtubules. Science, 2006, 312:1491–1495. 10.1126/science.1126551 16627697

[41] Zabotina O, Malm E, Drakakaki G. Identification and preliminary characterization of a new chemical affecting glucosyltransferase activities involved in plant cell wall biosynthesis. Mol Plant. 2008, 1:977–89. 10.1093/mp/ssn055 19825597

[42] Qi J, Wu B, Feng S, Lü S, Guan C, Zhang X, et al Mechanical regulation of organ asymmetry in leaves. Nat Plants, 2017, 3:724–733. 10.1038/s41477-017-0008-6 29150691

[43] Smith LG. Cytoskeletal control of plant cell shape: getting the fine points. Curr Opin Plant Biol, 2003, 6:63–73. 12495753 10.1016/s1369-5266(02)00012-2

[44] Szymanski DB, Wick M. Organized F-Actin is essential for normal trichome morphogenesis in Arabidopsis. Plant Cell, 1999, 11(12):2331–2347. 10.1105/tpc.11.12.2331 10590162 PMC144140

[45] Dong H, Pei W, Ren H. Actin fringe is correlated with tip growth velocity of pollen tubes. Mol Plant, 2012, 5:1160–1162. 10.1093/mp/sss073 22863760

[46] Riedl J, Crevenna AH, Kessenbrock K, Yu JH, Neukirchen D, Bista M, et al Lifeact: a versatile marker to visualize F-actin. Nat Methods, 2008, 5:605–607. 10.1038/nmeth.1220 18536722 PMC2814344

[47] Garcia V, Bres C, Just D, Fernandez L, Tai FW, Mauxion JP, et al Rapid identification of causal mutations in tomato EMS populations via mapping-by-sequencing. Nat Protoc, 2016, 11:2401–18. 10.1038/nprot.2016.143 27809315

