# Supplementary figures and images for: Spatiotemporal cytoskeleton organizations determine morphogenesis of multicellular trichomes in tomato
Source: PLoS Genet. 2019 Oct 4;15(10):e1008438. doi: 10.1371/journal.pgen.1008438 (PMC6812842; doi:10.1371/journal.pgen.1008438)

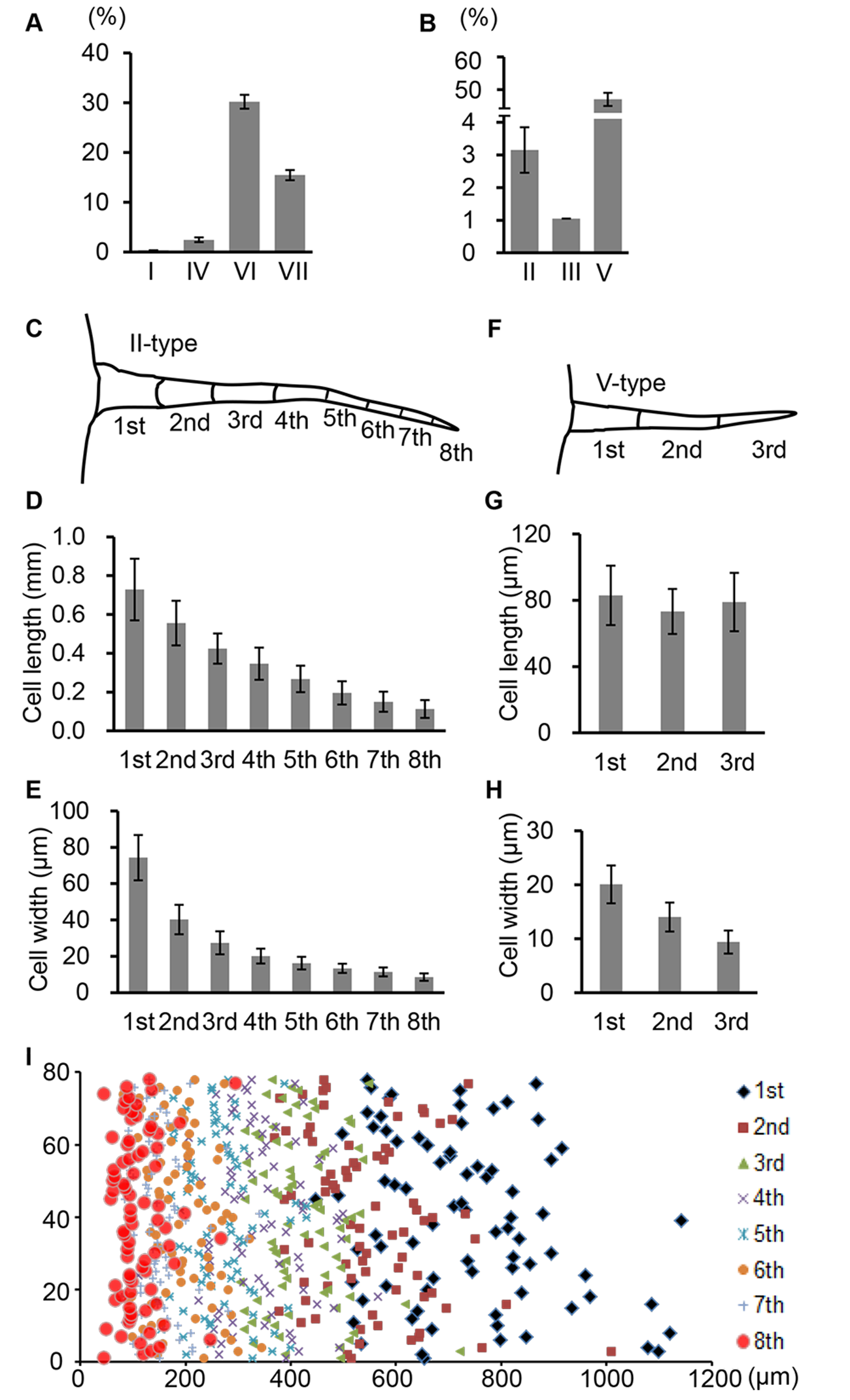

Supplement: S1 Fig — (A, B) The abundance of each trichome type. (C) Schematic diagram of the type II trichome. The basal cell that directly connects to the leaf epidermis is the first cell and the distal top cell is the eighth cell. (D, E) Cell length and cell width of each cell within type II trichome cell file. (F) Schematic diagram of the type V trichome. (G, H) Cell length and cell width of each cell within type V trichome cell file. (I) Scatter diagram of cell length of different cells within type II trichome cell file. Y-axis shows the number of trichomes used for measuring cell length. X-axis shows the cell length of different cell (μm). (TIF) [file pgen.1008438.s001.tif]

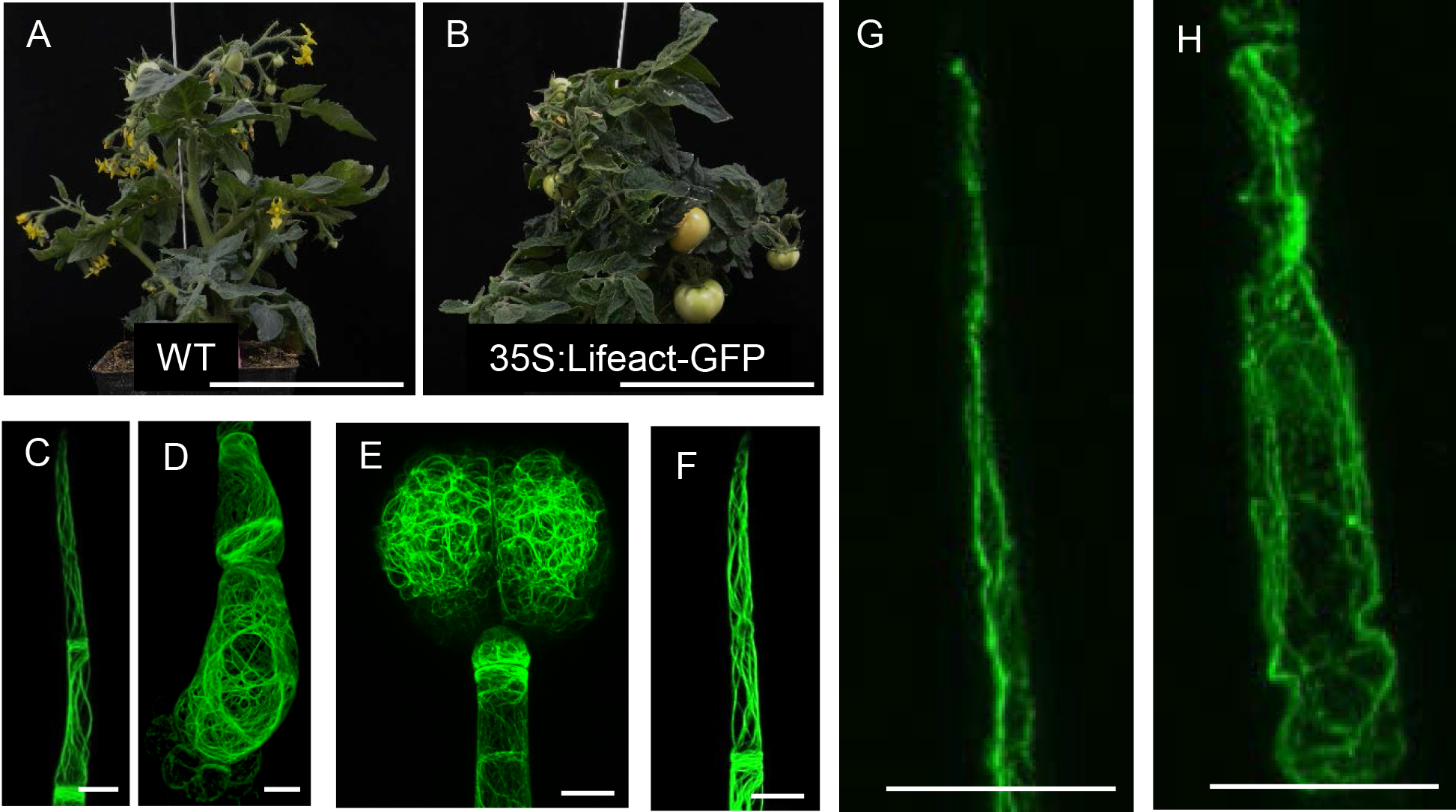

Supplement: S2 Fig — (A B) WT (A) and the normal transgenic plants (B). Bar:10 cm. (C D) The normal and abnormal actin alignment in transgenic plants with abnormal morphology. Bar:25 μm. (E F) The normal actin alignment in the head of the type VI trichome (E) and the top cell of type II trichom (E) in the normal transgenic plants. Bar:20 μm. (G H) Immuno-staining images of actin filaments in the top cell (G) and the stalk cell of trichomes (H) in WT using anti-actin. Bar: 25 μm. (TIF) [file pgen.1008438.s002.tif]

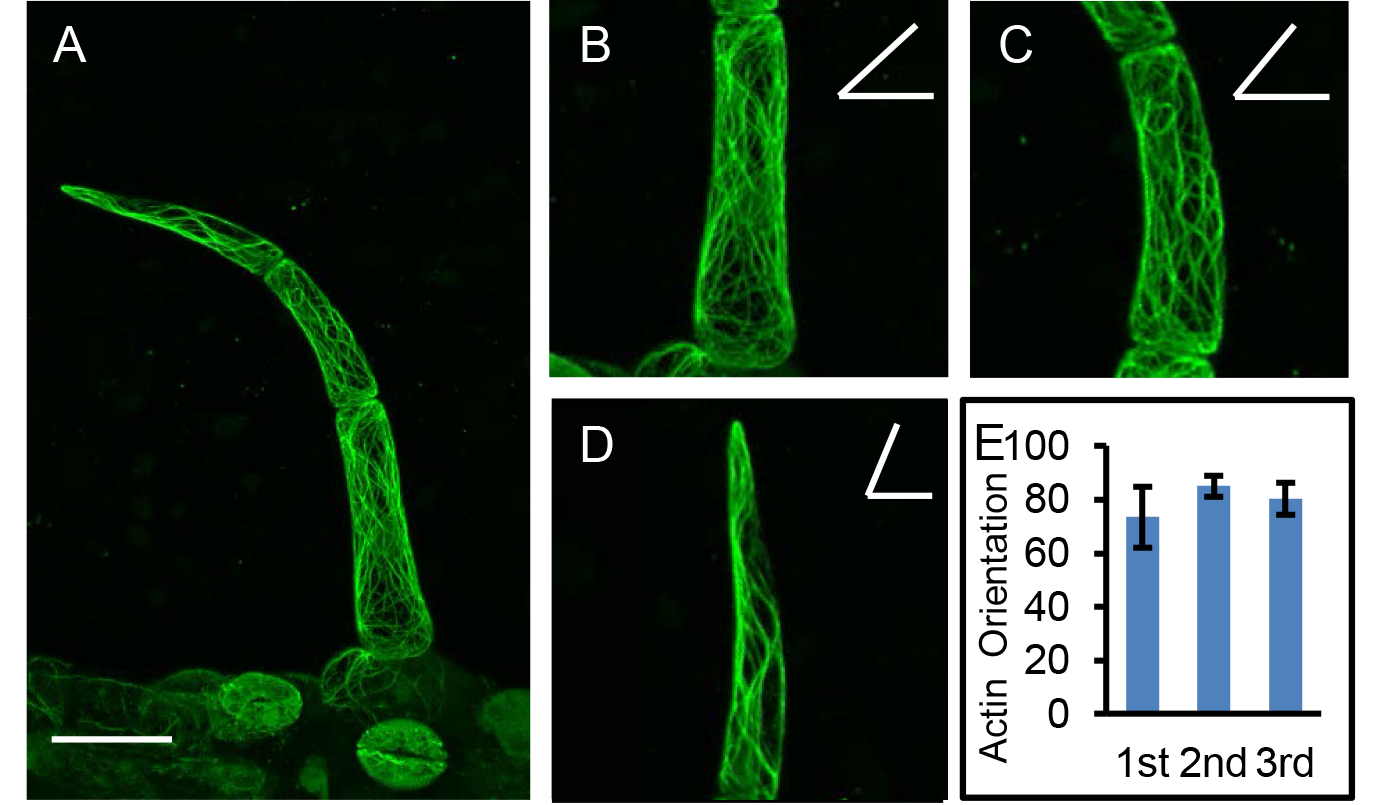

Supplement: S3 Fig — (A) A panoramic micrograph of actin organization in the type V trichome cell file. Bar: 50 μm. (B-D) Details of actin arrangement in each cell of the type V trichomes. E) Average orientation of cortical actin filaments in type V trichomes by Image J. (TIF) [file pgen.1008438.s003.tif]

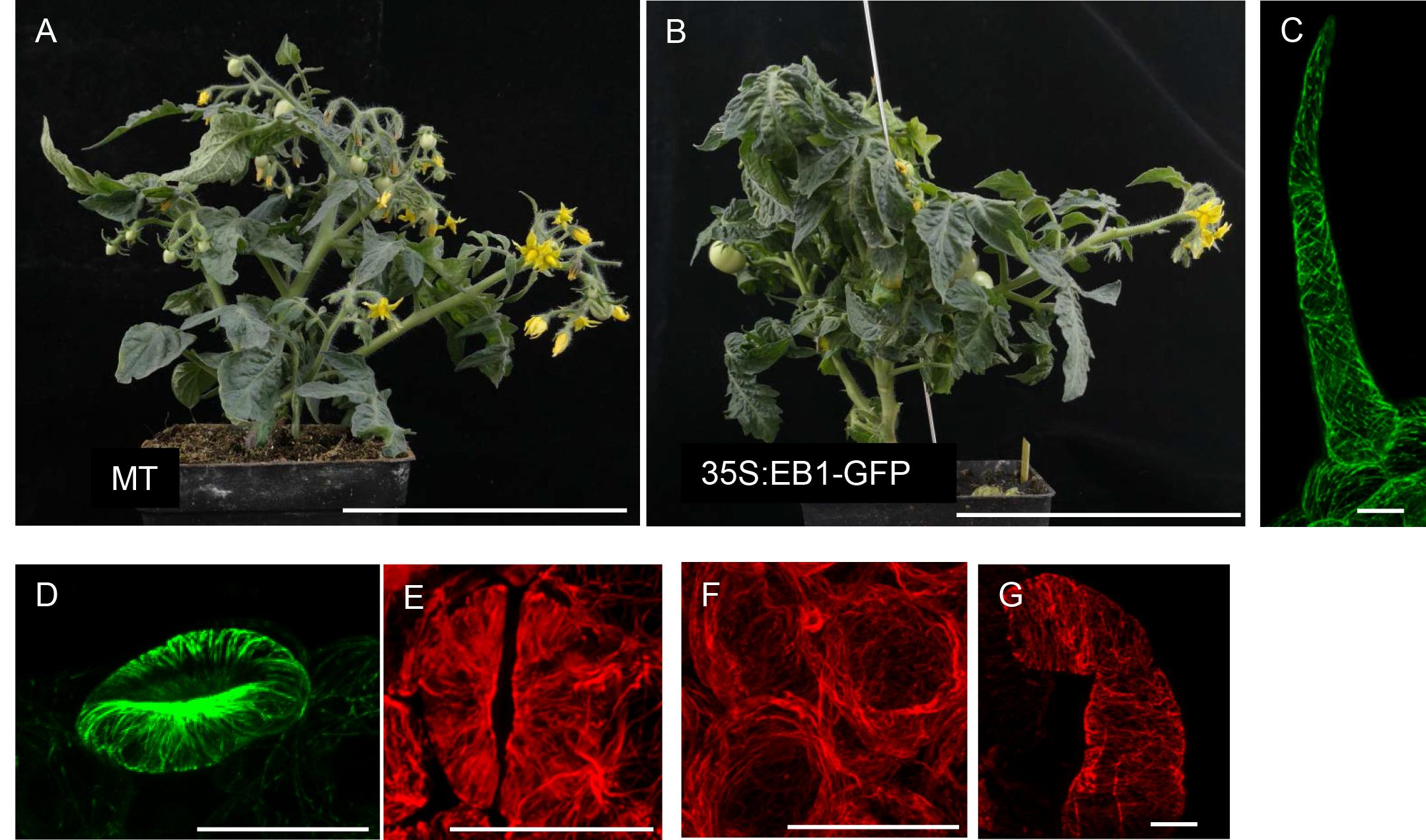

Supplement: S4 Fig — (A B) WT (A) and the transgenic plants (B); Bar: 10cm. (C D) The signal of EB1a-GFP in type V trichomes (C) and stomata (D). Bar: 20 μm. (E-G) Immuno-staining image of microtubules in the stomata (E), the mesophyll cells (F) and the stalk cell of trichomes (G) in WT using anti-tubulin. Bar:20 μm. (TIF) [file pgen.1008438.s004.tif]

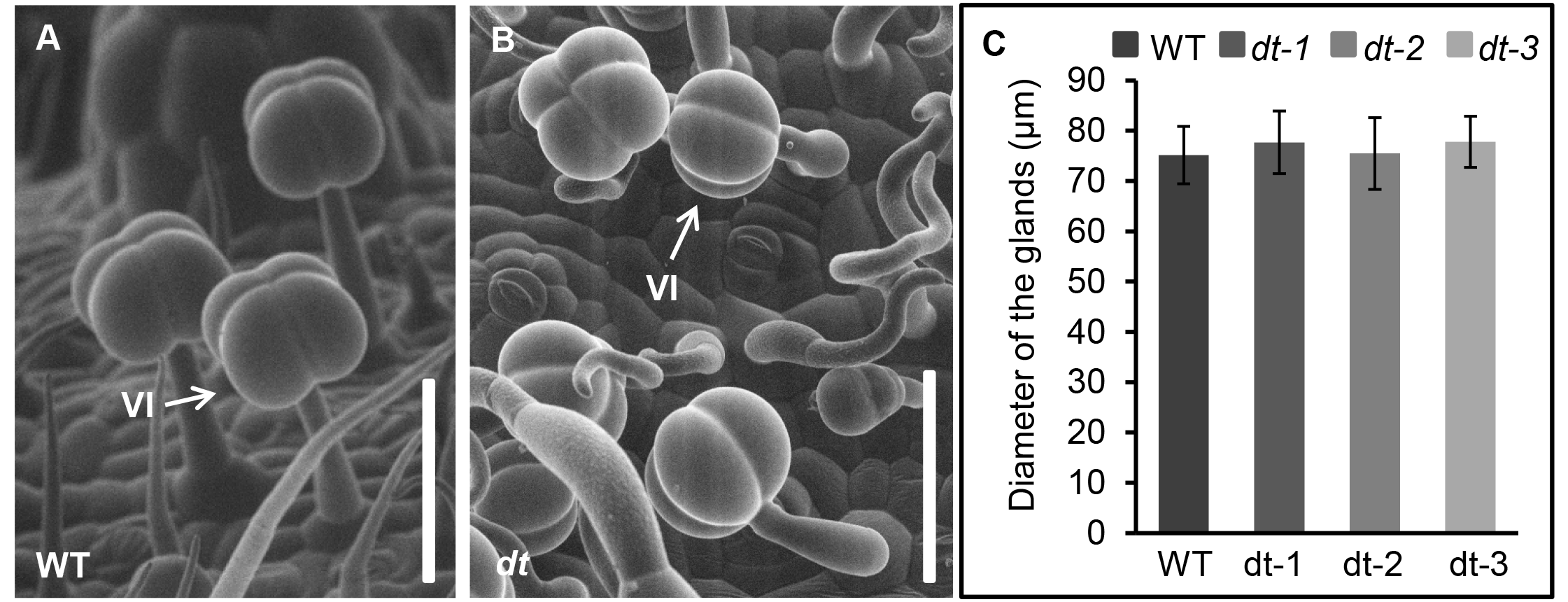

Supplement: S5 Fig — (A B) Phenotype of type VI glandular trichomes of WT and dt mutants by SEM. Bar: 100 μm. (C) Diameter of the gland heads of type VI trichomes. (TIF) [file pgen.1008438.s005.tif]

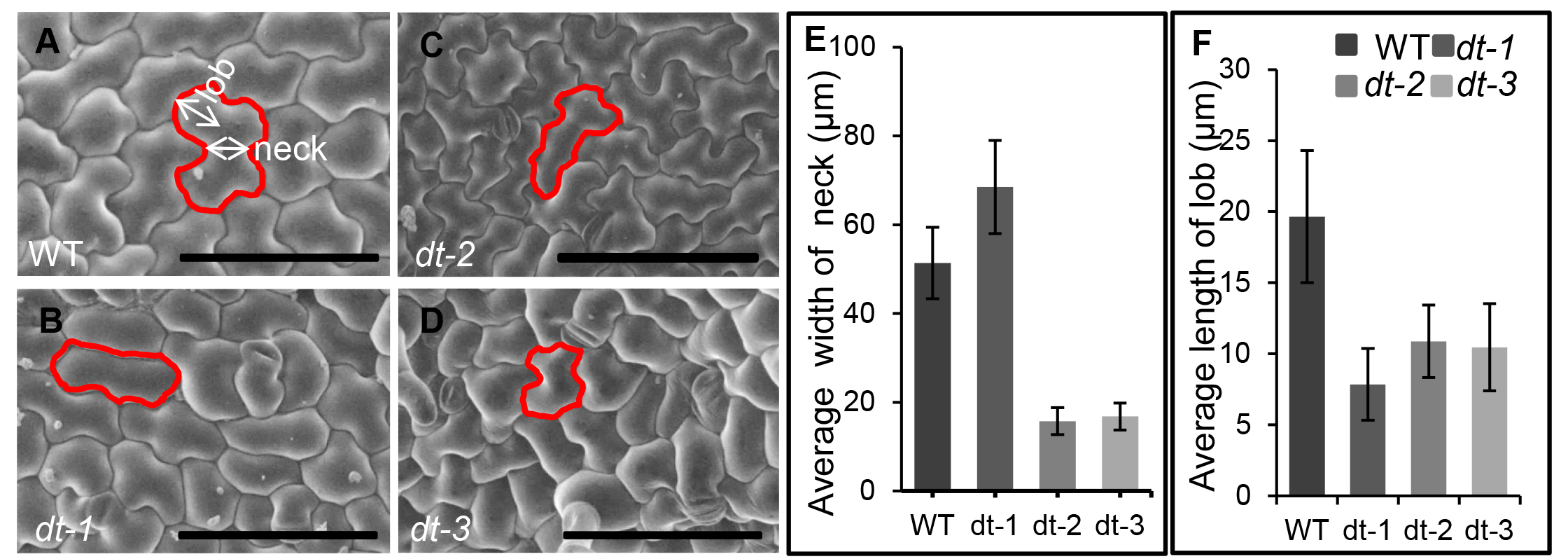

Supplement: S6 Fig — (A-D) SEM micrographs showing pavement cell shape in the WT (A) and dt mutants. (C-D). The lob and neck were shown in the (A). Bar: 200 μm. (E) Average width of the neck in the WT and dt mutants. (*P<0.1 and**P<0.01). (F) Average length of the lob in the WT and dt mutants. (*P<0.1 and**P<0.01) (TIF) [file pgen.1008438.s006.tif]

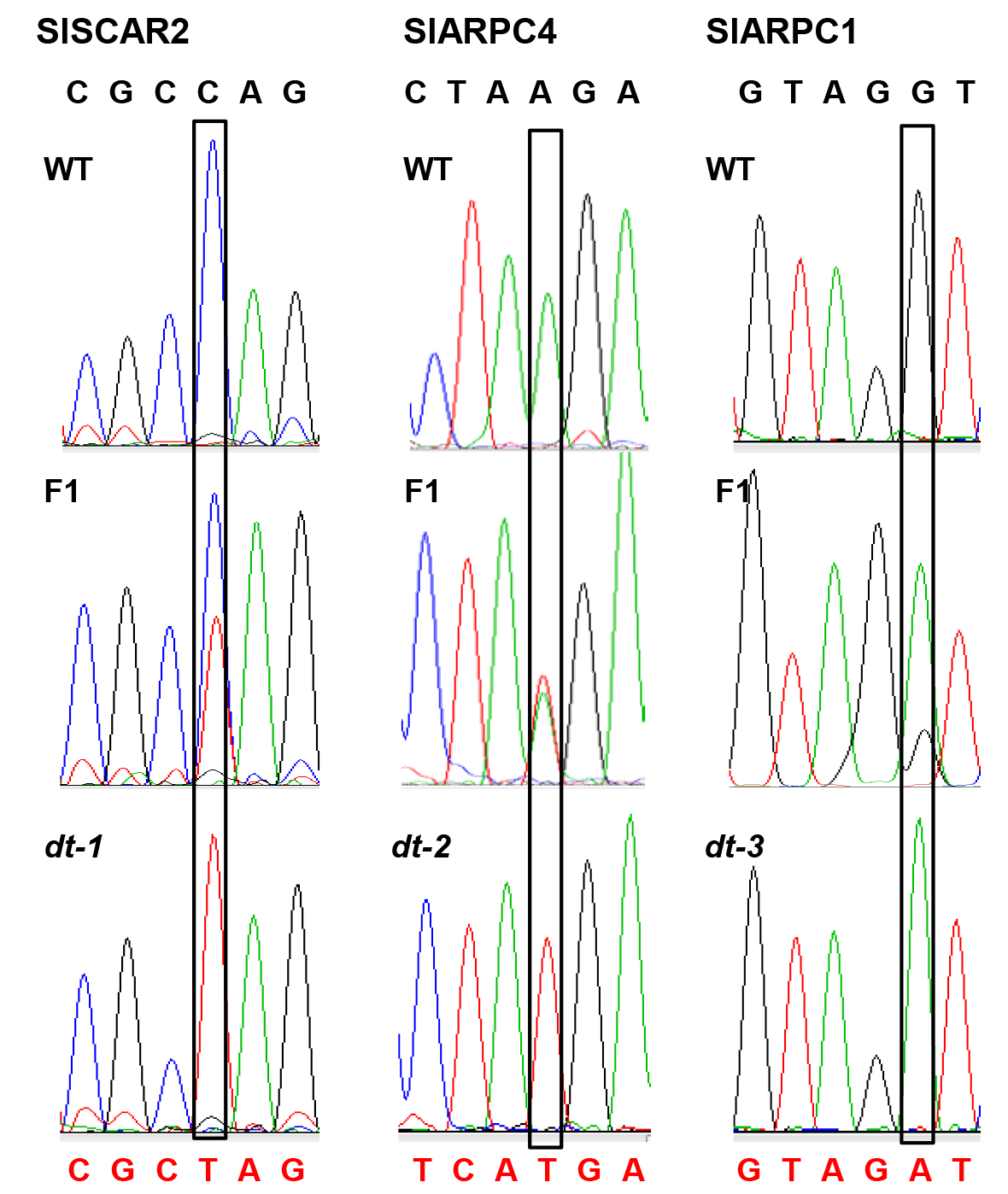

Supplement: S7 Fig — (TIF) [file pgen.1008438.s007.tif]

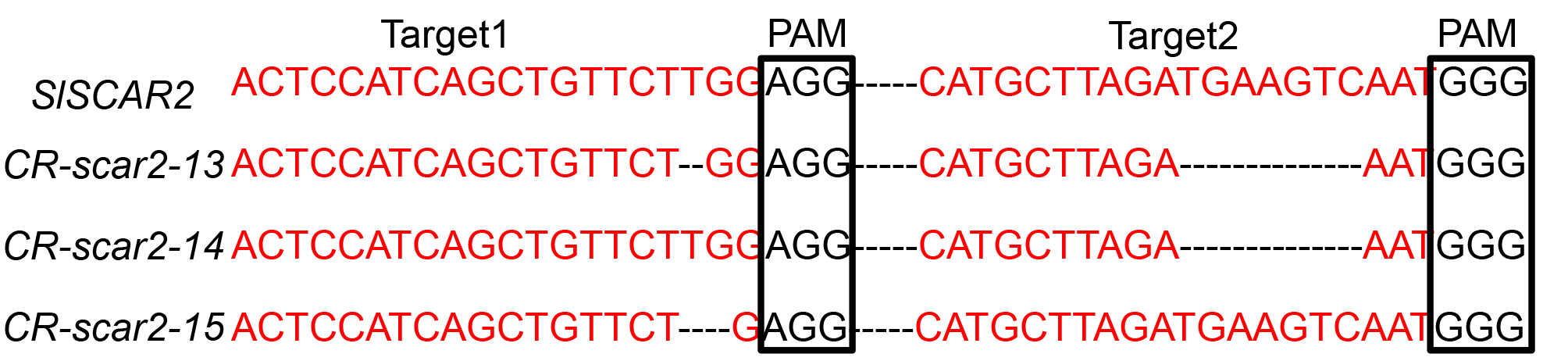

Supplement: S8 Fig — Allele sequences that were determined by sequencing are shown. (TIF) [file pgen.1008438.s008.tif]

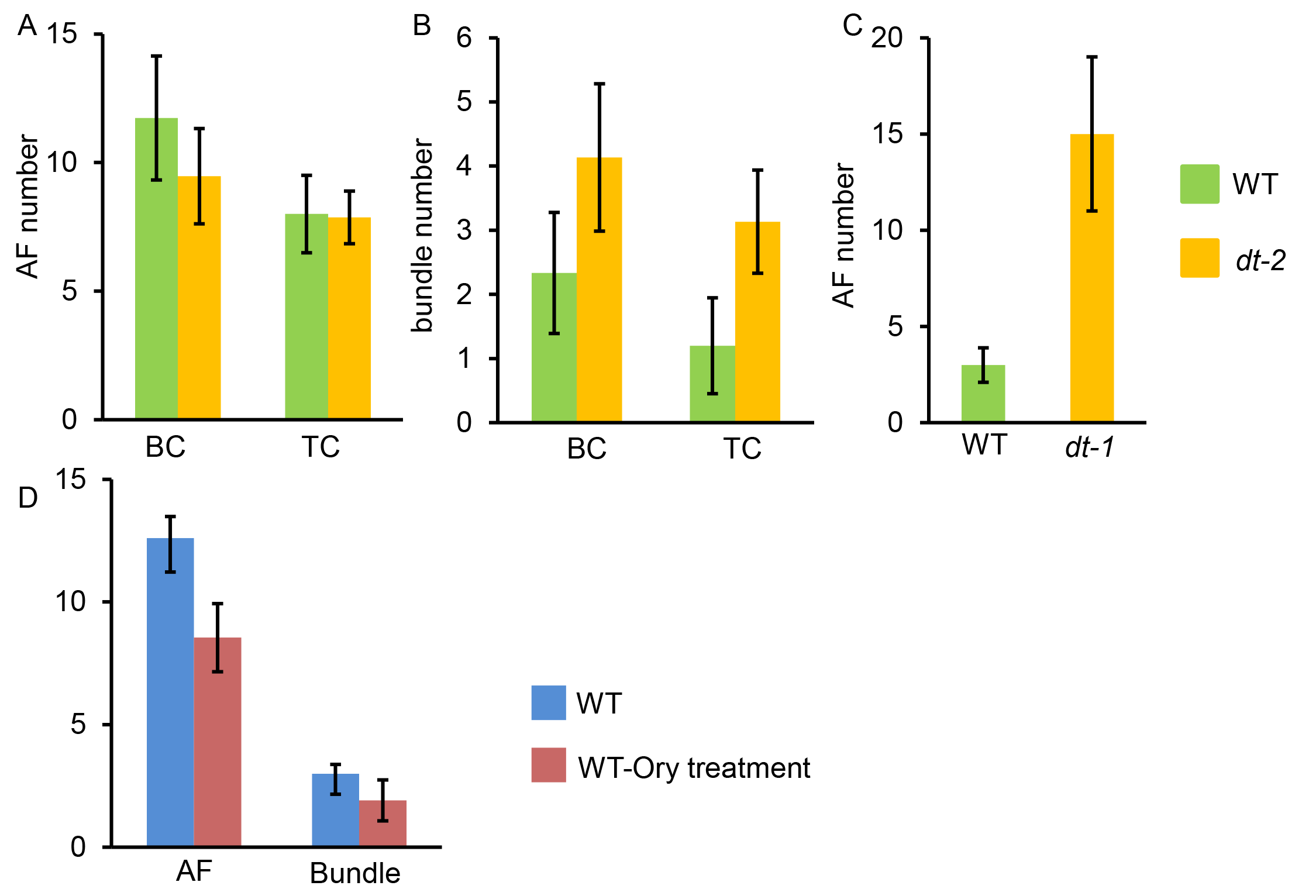

Supplement: S9 Fig — (A) The quantification of cortical actin filaments (AFs) in the basal cell (BC) and the top cell (TC) in the WT and dt-2. (B) The quantification of cortical actin cables in the basal cell (BC) and the top cell (TC) in the WT and dt-2. (C) The quantification of actin cables in the cytoplasm of the basal cell. (D) The quantification of the F-actin filaments and cables after Oryzalin treatment. (TIF) [file pgen.1008438.s009.tif]
